# A hybrid swarm intelligence algorithm for region-based image fusion

**DOI:** 10.1038/s41598-024-63746-w

**Published:** 2024-06-14

**Authors:** Rohit Salgotra, Amanjot Kaur Lamba, Dhruv Talwar, Dhairya Gulati, Amir H. Gandomi

**Affiliations:** 1 Faculty of Physics and Applied Computer Science, AGH University of Science & Technology, Kraków, Poland; 2 MEU Research Unit, Middle East University, Amman, Jordan; 3 Department of Electronics and Communication Engineering, Punjab Engineering College (Deemed-to-be University), Chandigarh, India; 4 Faculty of Engineering and IT, University of Technology Sydney, Ultimo, NSW 2007 Australia; 5 University Research and Innovation Center (EKIK), Óbuda University, Budapest, 1034 Hungary

**Keywords:** Multiple algorithms, Adaptivity, Hybridization, Naked mole rat algorithm, Image fusion, Electrical and electronic engineering, Computational science, Computer science

## Abstract

This paper proposes a novel multi-hybrid algorithm named DHPN, using the best-known properties of dwarf mongoose algorithm (DMA), honey badger algorithm (HBA), prairie dog optimizer (PDO), cuckoo search (CS), grey wolf optimizer (GWO) and naked mole rat algorithm (NMRA). It follows an iterative division for extensive exploration and incorporates major parametric enhancements for improved exploitation operation. To counter the local optima problems, a stagnation phase using CS and GWO is added. Six new inertia weight operators have been analyzed to adapt algorithmic parameters, and the best combination of these parameters has been found. An analysis of the suitability of DHPN towards population variations and higher dimensions has been performed. For performance evaluation, the CEC 2005 and CEC 2019 benchmark data sets have been used. A comparison has been performed with differential evolution with active archive (JADE), self-adaptive DE (SaDE), success history based DE (SHADE), LSHADE-SPACMA, extended GWO (GWO-E), jDE100, and others. The DHPN algorithm is also used to solve the image fusion problem for four fusion quality metrics, namely, edge-based similarity index ($Q^{AB/F}$), sum of correlation difference (*SCD*), structural similarity index measure (*SSIM*), and artifact measure ($N^{AB/F}$). The average $Q^{AB/F} = 0.765508$, $SCD = 1.63185$, $SSIM = 0.726317$, and $N^{AB/F} = 0.006617$ shows the best combination of results obtained by DHPN with respect to the existing algorithms such as DCH, CBF, GTF, JSR and others. Experimental and statistical Wilcoxon’s and Friedman’s tests show that the proposed DHPN algorithm performs significantly better in comparison to the other algorithms under test.

## Introduction

Over the last three decades, several natural phenomena have been used to formulate new mathematical generalizations for solving optimization problems including medical imaging^1–3^, robotics, business management, mathematical science^4,5^, segmentation^6–10^, clustering^11^, feature selection^12–16^, among others^17–19^. These algorithms are used because of their simple implementation and low computational complexity. Apart from that, the algorithms are faster in convergence and have fewer parameters and better exploration (*expl*) as well as exploitation (*expt*) properties. The algorithms include swarm intelligent algorithms (SIAs) and evolutionary algorithms (EAs). EAs consists of genetic algorithm (GA)^20^, memetic algorithm (MA)^21^, scatter search (SS)^22^, differential evolution (DE)^23^, stochastic fractal search (SFS)^24^, among others. These algorithms are among the earliest known algorithms in this domain and use the theory of evolution. SIAs, on the other hand, follow swarming and are based on social groupings such as bird flocking, colonies of insects, herds of animals, and others. Some of the major algorithms of this group include particle swarm optimization (PSO)^25^, symbiotic organisms search (SOS)^26^, sandpiper optimization algorithm (SOA)^27^, red fox optimization (RFO)^28^, golden eagle optimizer (GEO)^29^, grasshopper optimization algorithm (GOA)^30^, clouded leopard optimization (CLO)^31^, hermit crab shell exchange (HCSE)^32^, mud ring algorithm (MRA)^33^, seahorse optimizer (SHO)^34^, escaping bird search (EBS)^35^, and honey badger algorithm (HBA)^36^, among others.

Naked mole rat algorithm (NMRA) is a recently proposed SIA^37^ and is applied to many domain research problems. It takes inspiration from the worker-breeder relationship of NMR’s found in nature. It is based on the mating of NMR’s and consists of two parts. The first part of the algorithm is the worker phase which consists of arbitrary solutions to control *expl* operation, whereas the second part is the breeder phase which is intended for *expt* operation. The algorithm is simple in implementation, but for higher dimensions, it becomes very difficult to optimize the results^38^. Apart from that, the algorithm still suffers from poor *expl* due to less randomization in the worker phase and there are chances of stagnation as well.

In the current article, a new hybrid variant of NMRA is proposed. This newly proposed algorithm uses a combination of three new algorithms, namely HBA^36^, dwarf mongoose algorithm (DMA)^39^ and Prairie dog optimizer (PDO)^40^. All of these algorithms are added in a specific manner to see which algorithm fits the best for which set of iterations. In general, we have used only those equations from these algorithms, which fit the best for our proposed approach. Here DMA, PDO and HBA-based equations are added to the worker phase of NMRA for enhanced *expl* operation. The exploitation operation of the basic NMRA is found to be highly exploitative and is kept intact. In order to deal with the stagnation problems, we introduce a new stagnation phase. Apart from the added advantages, the algorithm is made adaptive by using six different mutation/*iw* operators, including chaotic^41^, exponential decreasing^42^, linearly decreasing, oscillating^43^, simulated annealing^44^, and random. All of these modifications sum up to formulate the new algorithm.

In addition, the proposed hybrid algorithm is applied to the real-world problem of image fusion of infrared (IR) and visible (VIS) images. Image fusion is a process of merging two or more images that are obtained from various sensors^45,46^. This is done to obtain a highly informative image that contains details which cannot be comprehended by analysing its source images independently. VIS images tend to have superior visual quality and precision background attributes. However, IR images have more resilience against varying light levels and environmental deterioration^47^. Therefore, this work employs the proposed algorithm as an optimization strategy to perform region-based image fusion of IR and VIS images. Here, DHPN is used to perform segmentation of the VIS image to extract its salient features. The derived features are utilized for weight map computation of both VIS and IR images. These weight map functions are further used to form the final fused image.

The major highlights of this work are presented as:

- The best-known equations of four new algorithms, namely PDO, DMA, HBA, and NMRA, are used to formulate a new multi-hybrid algorithm.
- The equations of PDO, DMA, and HBA are incorporated into the worker phase of NMRA, for improved exploration operation, whereas the exploitation phase is kept same.
- The parameters of the proposed algorithm are made self-adaptive in nature by using six different mutation/iw operators. These operators are chosen to perform better exploration and exploitation along with a balanced operation.
- A new stagnation phase is introduced to help the algorithm come out of local optima stagnation and find the near-optimal solution.
- To test the performance of the proposed algorithm, CEC 2005 and CEC 2019 benchmark problems are used. These are challenging problems, and any algorithm performing well on these problems can be considered as a potential candidate for future research.
- Apart from these benchmark datasets, the proposed algorithm is further used for the optimization of real-world IR and VIS images. In the current study, we perform region-based image fusion of both IR and VIS images using the proposed DHPN algorithm.

Excluding introduction, the paper has seven sections. The second section provides details about the major algorithms used for formulating the proposed algorithm and a summary of literature. Section three details the requirement of the proposal, basic motivation, and extended novelty of the proposed work. We provide detailed results of the numerical benchmarks in Sect. 4. We have used two sets of benchmarks, namely CEC 2005^48^ and CEC 2019 benchmarks^49^. For experimental tests, we have used various new and hybrid algorithms, including JADE^50^, LSHADE-SPACMA^51^, and success-history based adaptive DE (SHADE)^52^ among others. A detailed parametric study, along with dimension size and population size (pop size) variability, is also checked and tested to see the applicability of the proposed work for higher dimension problems. In Sect. 5, we have used the proposed optimization algorithm for region-based image fusion of IR and VIS images. In the sixth section, a detailed summary of the results is presented. This section also includes some drawbacks of the proposed algorithm, along with some future insights. Conclusions are drawn and some future directions are given in the final section.

## Basics of nature-inspired algorithms

This section provides details on the algorithms used as the basis for the proposed algorithm.

### Naked mole-rat algorithm

NMRA was developed using the data from naturally occurring mating behaviours of species of naked mole-rats (NMRs). NMRA is broken down into three phases. Apart from the introduction, the worker phase is known for *expl* operation, whereas the breeder phase is meant for *expt*. Both these phases are shared by practically all nature-inspired algorithms. The essential NMRA’s mathematical formulations are as follows:

*Initialization:* Initial NMR population is defined in this phase with dimensional vector *D* in [1, 2, ..., *n*] interval. Here, *n* stands for the best possible pop size and *D* means various parameter combinations of the test issue. Each NMR’s initialization is shown by:

1 $$\begin{aligned} N_{i,j}=N_{min,j}+r\times (N_{min,j}-N_{max,j}) \end{aligned}$$

where in the intervals [1, 2, ..., *n*] and [1, 2, ..., *D*], *i* and *j* are defined. $N_{min,j}$ is the lower boundary and $N_{max,j}$ is the upper boundary of the problem under investigation. *r* is the random number between [0, 1] with uniform distribution.

*Worker phase:* Here, the worker NMRs’ fitness is assessed, and according to the result, it may be qualified to join the breeder’s. A chance of mating exists if the worker NMRs join the breeder’s. Utilizing prior knowledge, the worker NMR solution is computed. The workers are generated using,

2 $$\begin{aligned} x_{i}^{t+1}=x_{i}^{t}+\lambda (x_p^t-x_q^t) \end{aligned}$$

where $o_i^t$ for the *t* *th* iteration is the solution of the *i* *th* worker, and $o_i^{t+1}$ is the new solution produced by leveraging the prior solution. The arbitrarily solutions selected are $o_i^t$, $x_p^t$ and $x_q^t$. The mating factor $\lambda$ has a uniform distribution and falls between [0, 1].

*Breeder phase:* To mate with the queen or continue breeding, NMRs in breeder groups must become more physically fit. The best breeders $n_{best}$ update their position according to the probability of breeding (*bp*). Here, it is important to keep in mind that the fitness of a certain section of breeders does not improve over time and, hence, is shifted to the worker group. The new solutions in this phase are given by

3 $$\begin{aligned} y_{i}^{t+1}=(1-\lambda )y_i^t+\lambda (n_{best}-y_i^t) \end{aligned}$$

where $y_i^t$ is the result of the *i* *th* breeder during the *t* *th* iteration, $\lambda$ denotes a parameter that regulates mating and $y_i^{t+1}$ is the new breeder produced in the subsequent iteration. Initially set to 0.5, the value of *bp* can be any number between [0,1].

### Grey wolf optimizer

As the name suggests, GWO is inspired by grey wolves. All the individuals have a strict dominance hierarchy categorized as beta, alpha, delta and omega wolves. The mathematical model of GWO is given as:

*Social hierarchy:* The appropriate solution for each generation is considered as alpha $(\alpha )$ wolf. Respectively, beta $(\beta )$ is the second and third is delta $(\delta )$; and the rest are omegas $(\omega )$.

*Encircling prey:* Grey wolf’s ability is to recognize prey and encircle it. Mathematically, this behaviour is formulated as

4 $$\begin{aligned}{} & {} D=|P.x_p^t-x^t| \end{aligned}$$

5 $$\begin{aligned}{} & {} x^{t+1}=x_p^t-R.D \end{aligned}$$

where *t* represents the iteration counter, $x_p^t$ and $x^t$ denote the position of prey and grey wolf, respectively. The values of *R* and *P* are as:

6 $$\begin{aligned} R=2a.r_1-a \end{aligned}$$

7 $$\begin{aligned} P=2.r_2 \end{aligned}$$

where $$a \in [2,0]$$ and $$r_1$$, $$r_2$$ $$\in$$ [0,1].

*Hunting:* It is done by $\alpha$, $\beta$ and $\delta$. Guided by $\alpha$, here $\beta$ and $\delta$ might also participate in hunting occasionally. Thus, the remaining two agents ($\omega$ wolves) will also participate in hunting, and their position will be decided according to the positions of $\alpha$, $\beta$, $\delta$. The mathematical expression used in this phase is:

8 $$\begin{aligned}{} & {} D_{\alpha }=|Y_1.x_{\alpha }-x|;\hspace{5pt} D_{\beta }=|Y_2.x_{\beta }-x|;\hspace{5pt} D_{\delta }=|Y_2.x_{\delta }-x| \end{aligned}$$

9 $$\begin{aligned}{} & {} o_1=x_{\alpha }-G_1.(D_{\alpha });\hspace{5pt} o_2=x_{\beta }-G_2.(D_{\beta });\hspace{5pt} o_3=o_1=x_{\delta }-G_3.(D_{\delta }) \end{aligned}$$

10 $$\begin{aligned}{} & {} x^{t+1}=\frac{o_1+o_2+o_3}{3} \end{aligned}$$

*Attacking the prey (expt phase):* At the very last stage, the prey is attacked as per the hunting mechanism. The stage of *a* first contains, 2 which is now been decreased to 0. It will continuously and correspondingly affect the parameter *A* as per the Eq. (6). Thus, the effect of the equation will affect the value *A*, which will be in the range of [−1,1]. As per the equation, the search for the next prey will definitely be done. Thus, for $|A|<1$, the wolves will forcefully attack.

*Searching prey (expl phase):* For the search for prey algorithm, gray wolves must diverge their directions, which means that it has two options. Mathematically, this includes, $A \le -1$ or $A > 1$. These two options will allow the algorithm to go further. In the situation of $|A|>1$ the wolves get separated and find new prey. *C* helps the algorithm to control *expl* by assigning random weights and hence following a random behaviour for avoiding local optima.

### Cuckoo search

CS draws inspiration from nature and can handle challenging optimization issues. The main inspiration for this method came from the parasitic behavior of the natural cuckoo species. CS consists primarily of global (*expl*) and local search (*expt*), to obtain the optimum solution.

*Global search phase:* This phase focuses on the *expl* phase of the CS algorithm and makes the assumption that a cuckoo only lays a single egg. With this supposition, a new solution $o_i^{t+1}$ was evolved, employing Lévy flights for the *i* *th* cuckoo. The solution is given by:

11 $$\begin{aligned} o_i^{t+1}=o_i^{t}+\alpha \hspace{2pt}\otimes Levy (\lambda ) (x_{best}-o_i^t) \end{aligned}$$

where $o_i^{t}$ denotes the solution of *i* *th* cuckoo at *t* *th* iteration, $o_i^{t+1}$ represents the next solution and $x_{best}$ is the current best solution. Here, an arbitrary component of the Lévy distribution’s flight model is applied. This part, which basically imitates the cuckoo species’ flight path, is characterized as:

12 $$\begin{aligned} Levy\sim \mu =t^{-\Phi },(1<\Phi \le 3) \end{aligned}$$

where $\Phi$ is the expected outcome of an event.

*Local search phase:* The algorithm’s *expt* actions are reflected in this phase. The solution $o_i^{t+1}$ is built in this phase on the basis of two random solutions $x_p^t$, $x_q^t$ from the total population. For this stage, the following equation will produce a solution:

13 $$\begin{aligned} x_{i}^{t+1}=o_i^t+\alpha \otimes (\epsilon )\otimes (x_p^t-x_q^t) \end{aligned}$$

where $$\epsilon \in [0,1]$$.

### Honey badger algorithm

Honey badger (HB) is a fluffy black and white mammal that lives in semideserts and rain forests in Southwest Asia, Africa, and the Indian subcontinent. HBA mimics a HB’s foraging behaviour. The HB either smells and digs to find food sources, or it follows a honey-guide bird. The first situation is referred to as digging and honey mode. Previously, it senses smell to roughly localise the prey; then chooses the best digging spot so as to capture it. In the latter form, it uses a honeyguide to locate a beehive directly.

*Algorithmic Steps:* Theoretically, HBA includes both the *expl* and *expt* stages.

Population = $$\begin{bmatrix} o_{11} &{}\quad o_{12} &{}\quad o_{13} &{}\quad ... &{}\quad o_{1D}\\ o_{21} &{}\quad o_{22} &{}\quad o_{23} &{}\quad ... &{}\quad o_{2D}\\ ... &{}\quad ... &{}\quad ... &{}\quad ... &{}\quad ... \\ o_{n1} &{}\quad o_{n2} &{}\quad o_{n3} &{}\quad ... &{}\quad o_{nD}\\ \end{bmatrix}$$

*i* *th* position of HB $o_i$ = $[o_i^1,o_1^2,....,o_i^D]$

1. Initialization phase: Set HBs and their locations to their initial values based on Eq. (14).
  14 $$\begin{aligned} x_{i}=lb_{i}+r_{1} * (ub_{i} - lb_{i}) \end{aligned}$$
  where $r_1 \in [0,1]$, $o_i$ is *i* *th* HBs position, $lb_i$ is lower bound and $ub_i$ is upper bound.
2. Intensity(I): This is the difference between prey and HB and is given by
  15 $$\begin{aligned}{} & {} I_i = r_2 * S/4\pi g_i^2 \end{aligned}$$
  16 $$\begin{aligned}{} & {} S=(o_i - o_{I+1})^2 \end{aligned}$$
  17 $$\begin{aligned}{} & {} g_i = x_{prey} -o_i \end{aligned}$$
  where S defines source strength and $g_i$ is the prey’s and *i* *th* HB’s distance.
3. Density Factor $(\alpha )$: To guarantee a transition from *expl* to *expt*, the density factor regulates randomization.
  18 $$\begin{aligned} \alpha = C*exp(-t/t_{max}) \end{aligned}$$
  where $t_{max}$ is the maximum iterations, $C \ge 2$
4. Escaping Local Optimum: The three steps that follow are utilized to leave local optima zones by employing a flag *H* that modifies the search for higher *expl*.
5. Updating Positions: It constitutes two phases;
6. Digging Phase: The intensity of the prey’s smell I, HB’s and the prey $d_i$ distance, and the influence of $\alpha$ are all important in the digging phase for HBs.
  19 $$\begin{aligned} x_{new}=x_{prey} + H*I*\beta *x_{prey} +H *\alpha *r_3*g_i * |cos(2\pi r_4) *[1-cos(2\pi r_5]| \end{aligned}$$
  where $x_{prey}$ signify the location of prey; $\beta$ $\ge$ 1 (default = 6) is HBs capability to search food; $g_i$ denotes the distance between the *i* *th* HB and prey; $r3, r4, r5 \in [0, 1]$.
7. Honey Phase: We can model the scenario where an HB pursues a honeyguide bird to a beehive as;
  20 $$\begin{aligned} x_{new} =x_{prey}+H*r_7*\alpha *g_i \end{aligned}$$
  where $x_{new}$ is the new position of HB, and $x_{prey}$ is prey location.

### Dwarf mongoose algorithm

The tiniest carnivore found in Africa is the dwarf mongoose (DM). Due to their territorial nature, DM’s frequently mark horizontal objects in their domain with their cheek and anal glands.

The suggested DMO algorithm consists of the DM’s compensatory behavioural adaptation. Limiting the size of the prey, social behaviour (babysitters), semi-nomadic living, and other adaptations are examples of compensatory behaviour. The model consists of DMs as scouts, alpha group, and babysitters. The DMO algorithm starts with initialising the candidate population of the DMs (X)

21 $$\begin{aligned} O = \begin{bmatrix} m_{1,1} &{}\quad m_{1,2} &{}\quad ... &{}\quad m_{1,D-1} &{}\quad m_{1,D}\\ m_{2,1} &{}\quad m_{2,2} &{}\quad ... &{}\quad m_{2,D-1} &{}\quad m_{2,D}\\ ... &{}\quad ... &{}\quad m_{i,j} &{}\quad ... &{}\quad ... \\ m_{n,1} &{}\quad m_{n,2} &{}\quad ... &{}\quad m_{n,D-1} &{}\quad m_{n,D}\\ \end{bmatrix} \end{aligned}$$

where *O* is the populations generated randomly, $m_{i,j}$ signifies the location of the *j* *th* dimension of the *i* *th* member, *n* and *D* denote the pop size and dimension, respectively.

The proposed DMO algorithm’s optimization processes are divided into three phases.

#### Alpha group

The alpha female ($\alpha$) is chosen according to the probability calculated for each population fitness.

22 $$\begin{aligned} \alpha =\frac{fit_i}{\sum _{i=1}^{n}fit_i} \end{aligned}$$

If *bs* represents the number of babysitters, then $n-bs$ gives the DMs in the alpha group. Every DM sleeps in the initial sleeping mound, which is set to $\phi$.

23 $$\begin{aligned} o_{I+1} = o_i +\phi *peep \end{aligned}$$

where $$\phi \in [-1, 1]$$

The sleeping mound is given as $sm_i$

24 $$\begin{aligned} sm_i=\frac{fit_{i+1} - fit_i}{\max \{fit_{i+1},fit_i\}} \end{aligned}$$

The sleeping mound’s average value is provided by;

25 $$\begin{aligned} \varphi =\frac{\sum _{i=1}^{n}sm_i}{n} \end{aligned}$$

Once the criterion is satisfied, DMO hops to the scout phase, where another source is assessed.

#### Scout group

The DMs are known to avoid returning to the former sleeping mound, so the scouts search for an adjacent one to ensure *expl*. This is modelled as

26 $$\begin{aligned} o_{I+1}= \left\{ \begin{array}{ll} o_i - CD*phi*rand*[o_i-\textbf{M}] &{}\quad \text{ if } \varphi _{i+1}>\varphi _i \\ o_i + CD*phi*rand*[o_i-\textbf{M}] &{}\quad \text{ else } \end{array} \right. \end{aligned}$$

where *rand* denotes a random number in [0, 1], CD = $\Big (1-\frac{iter}{Max_{iter}}\Big )^{\Big (2\frac{iter}{Max_{iter}}\Big )}$ regulates the collective-volatile movement of DM’s, and is decreased linearly. $\textbf{M} = \sum _{i=1}^{n} \frac{o_i*sm_i}{o_i}$ determines the movement of the DM towards the new food source.

#### The babysitters

The group’s subordinate members who stay with the babies are typically rotated on a regular basis as the babysitters, allowing the alpha to guide the other group members. She usually comes back for nursing in the afternoon/evening. Total babysitters vary with pop size; and have an impact on DMO by decreasing the pop size according to the preset percentage. The exchange parameter helps reset the information between previous and current family members.

### Prairie dog optimization algorithm

Prairie dogs (PDs) are quite sociable and prefer to live together underground in large colonies. A colony usually houses 15-26 family units or coteries, with each coterie residing into its respective ward. The functionality and complexity of the subunits of a colony are same irrespective of the colony’s size.

In PDO^40^, PD populations are search agents, whereas their location represents the possible solution. The mathematical model of PDO is described below:

#### Initialization

Consider *n* number of PD in a coterie where each PD belongs to *m* coterie. The location of all coteries in a colony can be represented by a matrix *CO* given as:

27 $$\begin{aligned} CO = \begin{bmatrix} CO_{1,1} &{}\quad CO_{1,2} &{}\quad ... &{}\quad CO_{1,D-1} &{}\quad CO_{1,D}\\ CO_{2,1} &{}\quad CO_{2,2} &{}\quad ... &{}\quad CO_{2,D-1} &{}\quad CO_{2,D}\\ ... &{}\quad ... &{}\quad CO_{i,j} &{}\quad ... &{}\quad ... \\ CO_{m,1} &{}\quad CO_{m,2} &{}\quad ... &{}\quad CO_{m,D-1} &{}\quad CO_{m,D}\\ \end{bmatrix} \end{aligned}$$

where $CO_{i,j}$ denotes the *j*th dimension of the *i*th coterie. The location of all the PDs in a coterie can be represented by:

28 $$\begin{aligned} LO = \begin{bmatrix} LO_{1,1} &{}\quad LO_{1,2} &{}\quad ... &{}\quad LO_{1,D-1} &{}\quad LO_{1,D}\\ LO_{2,1} &{}\quad LO_{2,2} &{}\quad ... &{}\quad LO_{2,D-1} &{}\quad LO_{2,D}\\ ... &{}\quad ... &{}\quad LO_{i,j} &{}\quad ... &{}\quad ... \\ LO_{n,1} &{}\quad LO_{n,2} &{}\quad ... &{}\quad LO_{n,D-1} &{}\quad LO_{n,D}\\ \end{bmatrix} \end{aligned}$$

where $LO_{i,j}$ denotes the *j*th dimension of the *i*th PD such that $n \le m$. If *U*(0, 1) denotes a random number with a uniform distribution, then each coterie and PD location is given by:

29 $$\begin{aligned} CO_{i,j}=U(0,1) \times (UB_j -LB_j)+LB_j \end{aligned}$$

30 $$\begin{aligned} LO_{i,j}=U(0,1) \times (ub_j -lb_j)+lb_j \end{aligned}$$

where $ub_j=\frac{UB_j}{m}$ and $lb_j=\frac{LB_j}{m}$, such that $UB_j$ and $LB_j$ represent the upper and lower bounds of the *j*th dimension, respectively.

#### Fitness function evaluation

The value of fitness function of each PD location in a coterie is calculated by feeding the optimal solution to the fitness function defined as

31 $$\begin{aligned} fit(LO) = \begin{bmatrix} fit_1(LO_{1,1} &{}\quad LO_{1,2} &{}\quad ... &{}\quad LO_{1,D-1} &{}\quad LO_{1,D})\\ fit_2(LO_{2,1} &{}\quad LO_{2,2} &{}\quad ... &{}\quad LO_{2,D-1} &{}\quad LO_{2,D})\\ fit_n(LO_{n,1} &{}\quad LO_{n,2} &{}\quad ... &{}\quad LO_{n,D-1} &{}\quad LO_{n,D})\\ \end{bmatrix} \end{aligned}$$

At each iteration, the fitness function values are evaluated for all PDs and stored as $(n\times 1)$ matrix. Here, each fitness value represents the food source quality, the ability to build new burrows, and the apt response to the predators.

#### Exploration

The exploration operation is carried in $0<iter<\frac{T}{4}$ and $\frac{T}{4}\le iter<\frac{T}{2}$ intervals. The first step in this phase is the movement of PDs of a coterie from the ward in search of food. The position updating for the search can be expressed as

32 $$\begin{aligned} LO_{i+1}^{j+1}=gLO_{i,j}^{Best}-ecLO_{i,j}^{Best} \times \epsilon - CLO_{i,j} \times Levy(n) \ \forall \ 0<iter<\frac{T}{4} \end{aligned}$$

where $gLO_{i,j}^{Best}$ represents the globally best solution so far, $\epsilon$ denotes the food source alarm and Lévy(n) represents the Lévy(n) distribution. Here, $ecLO_{i,j}^{Best}$ signifies the effect of current best solution which is defined as

33 $$\begin{aligned} ecLO_{i,j}^{Best}=gLO_{i,j}^{Best} \times \tau + \frac{LO_{i,j}\times mean(LO_{n,m})}{gLO_{i,j}^{Best}\times (UB_j-LB_j)+\tau } \end{aligned}$$

where $\tau$ signifies the individual PD position difference. Also, the collective impact of all the PDs in the colony, $CLO_{i,j}$, is given by

34 $$\begin{aligned} CLO_{i,j}=\frac{gLO_{i,j}^{Best}-rLO_{i,j}}{gLO_{i,j}^{Best}+\tau } \end{aligned}$$

where $rLO_{i,j}$ is the random position of the *i*th PD in the *j*th dimension.

The second step is to evaluate the food quality along with the digging strength in order to build new burrows. The position updating for the building of a burrow can be expressed as

35 $$\begin{aligned} LO_{i+1}^{j+1}=gLO_{i,j}^{Best} \times rLO \times DS \times Levy(n) \ \forall \ \frac{T}{4}\le iter<\frac{T}{2} \end{aligned}$$

where *DS* denotes the digging strength as defined below

36 $$\begin{aligned} DS=1.5 \times s \times \left( 1-\frac{iter}{Max_{iter}}\right) ^{\left( 2\frac{iter}{Max_{iter}}\right) } \end{aligned}$$

where *s* can be either -1 or 1 according to the odd or even current iteration, respectively.

#### Exploitation

In PDO, the exploitation mechanism is utilised to focus the search on promising locations identified in the previous phase. It is implemented according to the equations given below

37 $$\begin{aligned}{} & {} LO_{i+1}^{j+1}=gLO_{i,j}^{Best}-ecLO_{i,j}^{Best} \times \rho - CLO_{i,j} \times rand \ \forall \ \frac{Max_{iter}}{2}\le iter<3\frac{Max_{iter}}{4} \end{aligned}$$

38 $$\begin{aligned}{} & {} LO_{i+1}^{j+1}=gLO_{i,j}^{Best} \times PR \times rand \ \forall \ 3\frac{Max_{iter}}{4} \le iter \le Max_{iter} \end{aligned}$$

where $\rho$ represents the food source quality, *rand* is a random number between 0 and 1 and *PR* is the effect of predators which can be expressed as

39 $$\begin{aligned} PR=1.5 \times (1-\frac{iter}{Max_{iter}})^{(2\frac{iter}{Max_{iter}})}. \end{aligned}$$

### Summary of literature

In the above sections, we have introduced the basic algorithms used for the formulation of the proposed algorithm. Apart from the introduction of a new algorithm, the proposed algorithm has also been applied to CEC benchmarks and image fusion problems. A recent list of applications with some modified algorithms in presented in Table 1.

**Table 1:** Recent literature on algorithms and their applications.

Reference no.	Optimization algorithm used	Application
Özbay^4^	Modified seahorse optimization algorithm	Engineering design problems
Gharehchopogh and Ibrikci^6^	Improved African vultures optimization algorithm	Multi-level thresholding image segmentation
Krishna et al.^11^	K-means and PSO algorithm	Clustering
Eluri and Devarakonda^12^	Chaotic binary pelican optimization algorithm	Feature selection
Eluri and Devarakonda^14^	Binary flamingo search algorithm and genetic algorithm	Feature selection
Abed-Alguni et al.^15^	Opposition-based sine cosine optimizer	Feature selection
Abed-Alguni et al.^16^	Improved binary djaya algorithm	Feature selection
Gharehchopogh et al.^17^	Dynamic harris hawk optimization algorithm	Botnet detection in IoT
Cheng and Prayogo^26^	Symbiotic organisms search optimization algorithm	Engineering design problems
Kaur et al.^27^	Sandpaper optimization algorithm	Engineering design problems
Mohammadi-Balani et al.^29^	Golden eagle optimizer	Engineering design problems
Saremi et al.^30^	Grasshopper optimization algorithm	Structural design problems
Trojovská et al.^31^	Clouded leopard optimization algorithm	Engineering design problems
Salgotra et al.^38^	Hybrid algorithm	Multi-level image thresholding
Shahdoosti and Tabatabaei^53^	Ant colony optimization algorithm	MRI and pet/spect image fusion
Panguluri and Mohan^54^	PSO algorithm	Thermal and VIS image fusion
Oliva et al.^55^	Electromagnetism optimization	Multi-level thresholding

A more detailed review of region-based image fusion is given in Sect. 5. The literature discussed in this section provides some insights on the applicability of the recently introduced algorithms on image segmentation problems. A major research gap in most of the works discussed above is in the implementation part of the algorithms. It has been found in most of the literature that the algorithms proposed are either simple modifications in the basic algorithm, enhancements in the parameters or merely an adaptation in a certain section of the algorithm. There is very limited work on the actual modification aspects, or mainly hybridization of algorithms pertaining to image thresholding and segmentation problems. So in the present work, a multi-hybrid algorithm with adaptive properties is proposed. In the next section, extensive details on how the proposed algorithm is formulated and every minute detail on why’s and how’s of this proposal are formulated.

## The proposed approach

Among the recently introduced algorithms, NMRA has been found to provide amazing *expl* and *expt* capabilities. The algorithm is highly reliable when compared with respect to the recently introduced CS, GWO, WOA and other algorithms. A comparison with the hybrid and enhanced versions shows that the algorithm has certain disadvantages too, and new improvements are required to make the algorithm self-sufficient in itself. One of the major disadvantages is the prevalence of poor *expl* operation of NMRA, which has been proved and highlighted in various recently introduced enhanced versions of NMRA^38^. It was analysed that due to less randomness in the solution space of the worker phase, and hence local optima stagnation. However, it can be improved by the addition of new prospective equations in the general working phase of the algorithm, thus, enhancing its global search properties. Also, parameters need to be enhanced, and self-adaptivity must be ensured so that no amendments are required. Based on this and the added advantages of new equations inspired by DMA, HBA, PDO, CS and GWO, a new multi-hybrid algorithm namely Dwarf Honey Parairie Naked mole-rat (DHPN) algorithm is proposed. The major highlights of this algorithm are

- Follows the basic structure of NMRA, and new modified equations are included in the worker phase. For the first one-fourth of iterations, basic NMRA equations are used, PDO-inspired equations are used for the second one-third of the iterations, for the third one-fourth of the iterations, DMA and for the final phase of iterations HBA, HBA-based equations are used.
- Equations of both CS and GWO are used in a hybrid manner, inspired by self-adaptive cuckoo search algorithm (SACS)^56^, and are meant for updating the whole solution set if the algorithm gets stuck. That is, if the solution quality doesn’t improve for certain iterations, the stagnation phase is activated and hence serves as the best player for avoiding local optima stagnation.
- For adding self-adaptive properties, six different mutation operators are added to the basic random parameters of the DHPN algorithm. All these mutation operators have algorithms that have been exploited in the literature and more details are presented in subsequent subsections.

A detailed discussion of what’s, how and why’s of the requirement of the proposal is given in the next subsections.

### What is the requirement of the proposal

In the recent literature, it has been found that new algorithms are being proposed and added to the expanding literature. However, the performance evaluation of these algorithms is limited to certain basic optimization algorithms only, and a comparison with respect to recent hybrid algorithms is missing. Even in some cases, the comparison is present, but enhanced search shows that there isn’t any significant improvement in the performance of newly proposed algorithms. Another thing that has been pointed out in the literature is the prevalence of problem-based modification as pointed out by the no free lunch theorem (NFL)^57^. According to NFL, no algorithm is perfect for all problems and user-based enhancements are required to fit it to a certain domain research problem. This is because every optimization problem consists of a different scenario, including variable dimension size, constrained or unconstrained nature, computational complexity, scalability, and others. The total number of local minima also poses a significant challenge to solving these problems. This provides us with enough evidence and motivation for the proposal of new algorithms. Apart from the generalized scenarios, NMRA also has the drawback of poor *expl*, and user-based modification is required to improve its *expl* properties. Why NMRA has poor *expl*?, it is because of the lesser random nature of the solutions and the problem of stagnation^38^. Apart from these problems, the basic algorithm uses only random initialization of parameters, which makes it very difficult to identify which set of combinations fits the best for the used parameters. A simple constant value is employed in most cases, thus following constant step sizes and restricting the search of the algorithm to particular regions. Adding a new combination of mutation operators using self-adaptive formulations makes the algorithm follow variable steps and provide excellent *expl* and *expt* properties. This provides us with enough motivation to propose a new prospective algorithm. In the present case, we present a novel DHPN algorithm based on the added properties of different algorithms and mutation operators. Modifications to the new equation are added in the global search or worker phase. The breeder phase is kept as such, and no equations are modified. More details on how the modifications have been added are provided in the next subsections.

### Motivation behind the proposal

Based on the rise in hybridization among optimization researchers, some of the most successful results for combinatorial and practical problems have been achieved through hybrid algorithms. One of the earliest instances of algorithm combination involved simulated annealing, genetic algorithms, tabu search, descent local search, and evolutionary algorithms, yielding notable outcomes^58^. The taxonomy of heuristic algorithms comprises hierarchical and flat classifications. The hierarchical level reduces the number of classes, while the flat level arranges techniques in an arbitrary order. In hierarchical taxonomy, low-level and high-level hybridization are distinguished. Low-level hybridization involves replacing a portion of the algorithm with another, whereas high-level hybridization involves self-contained algorithms with no direct internal relationship. Further, low-level and high-level hybridizations are categorized as Relay and Teamwork hybridization. Relay hybridization employs multiple algorithms sequentially, with the output of one serving as the input for the next, akin to a pipelined operation. On the other hand, Teamwork hybridization employs several parallel cooperating algorithms, each conducting an independent search. Overall, these characteristics classify hybrid algorithms into four types^58^.

- LRH : Low-level relay hybrid
- LTH : Low-level teamwork hybrid
- HRH : High-level relay hybrid
- HTH : High-level teamwork hybrid

In LRH, a single-solution algorithm incorporates an embedded algorithm. This approach is exemplified in the combination of simulated annealing with local search for solving the travelling salesman problem^59^. LRH models typically balance exploration and exploitation operations by utilizing different algorithms. However, since heuristics are often stronger in exploration than in exploitation, in LTH, one algorithm handles exploration while another tackles exploitation. For instance, Chu et al.^60^ proposed an LTH algorithm by integrating a generalized GA with tabu search for mutation and hill-climbing for crossover operations. On the other hand, HRH evaluates self-sufficient algorithms sequentially. In a study by^61^, tabu search and simulated annealing were employed to enhance the population generated by a GA over iterations. In HTH, several self-sufficient algorithms collaborate in parallel, each contributing to the search operation. For example, Cohoon and Hegde^62^ applied a GA as the base algorithm, with sections of the population being evaluated using simulated annealing, genetic programming, evolution strategy, and tabu search to improve overall performance.

In this study, we propose a novel algorithm based on the LTH model. Our approach combines multiple algorithms to execute the exploration operation, with each algorithm providing distinct solutions. These solutions are subsequently evaluated by another algorithm during the exploitation phase. Specifically, we employ equations inspired by PDO, DMA, HBA, and NMRA for the enhanced worker phase (exploration), while the exploitation phase leverages breeder equations from NMRA. Within a single iteration, one set of equations, inspired by one algorithm, conducts the exploration operation, while the other set handles exploitation. After a predefined number of iterations, the first algorithm is substituted with a new one, gradually transitioning to a multi-hybrid approach. By utilizing a combination of algorithms and employing a teamwork strategy, our method falls within the LTH model. This approach, integrating two or more algorithms, facilitates effective exploration and exploitation, enhancing the overall optimization process.

In a generalized algorithm, the aim is to conduct extensive exploration in the initial phases, gradually transitioning towards increased exploitation, and finally emphasizing extensive exploitation in the later stages. In our approach, we utilize equations inspired by DMA, HBA, PDO, and NMRA for iterative search, while employing CS-GWO-based equations for the stagnation phase. Before implementing the multi-hybrid algorithm, a preliminary study is essential to determine which algorithms excel in exploration, exploitation, and facilitating the transition between the two. Thus, our study proposes a multi-hybrid algorithm that incorporates the most effective equations for exploration and exploitation. We divide the iterations into four distinct phases, with each phase employing a specific set of equations from one of the algorithms. Additionally, we consider the parameters of each algorithm to optimize the performance of the proposed new algorithm. This approach aims to leverage the strengths of different algorithms while ensuring an effective balance between exploration and exploitation throughout the optimization process.

### The proposed approach

As already stated, the DHPN algorithm has the added advantages of PDO, DMA, HBA, and CS/GWO inspired self-adaptive cuckoo search (SACS)^56^. In this section, we deal with the detailed study of the proposal of the DHPN algorithm. It consists of five different phases, where initialization is the first phase, and the second worker phase incorporates some major changes and is the main phase. This phase provides excellent *expl* properties and incorporates major details using all the new algorithms under consideration. The next phase is the breeder phase, which has equations of NMRA. The fourth phase is meant for the selection of the best individuals over the course of iterations. A new phase inspired by the SACS algorithm is incorporated as a stagnation phase and is meant to improve the local search capabilities and also help to counter the local optima problems. A new subsection is added to analyse the parameters of the proposed algorithm. Details about the algorithm are presented below:

#### Initialization of the proposed DHPN algorithm

Initialization stands for the generation of randomized solutions for a *D* dimensional problem within a certain range defined by $N_{min,j}$ lower and $N_{max,j}$ upper bounds of the problem. The mathematical formulation is given by

40 $$\begin{aligned} N_{i,j}=N_{min,j}+r\times (N_{min,j}-N_{max,j}) \end{aligned}$$

where $$j \in [1,2,..., D]$$, $$i \in [1,2,..., n]$$, and $$r \in [0,1]$$.

#### Worker phase

The second and most interesting phase of the DHPN algorithm is the worker phase. It is for better *expl* properties and forms the core of the proposed algorithm. This phase is divided into four sub-phases.

a) Phase I: For Iterations. $\le$ $t_{max}/4$. The first phase of the worker group follows the same equations as used in the basic NMRA. These equations are based on two random solutions initialized within the search space. The two solutions are random, which makes the algorithm highly diverse in nature. This phase is mainly meant for explorative tendencies, and due to the presence of diverse solutions, we can achieve it conveniently. The mathematical equation is given by

41 $$\begin{aligned} x_{i}^{t+1}=x_{i}^{t}+\lambda (x_p^t-x_q^t) \end{aligned}$$

where $\lambda$ is a self-adaptive parameter analysed in consecutive subsections.

b) Phase II: For $t_{max}/4$ < iterations $\le$ $t_{max}/2$. This section uses the PDO^40^ algorithm for dedicated *expl* operation and is specifically meant for extensive global search. This search operation is to find solutions in close vicinity and in particular sections of the search space. The strategy is modelled using two equations of the PDO and is mathematically given by

42 $$\begin{aligned}{} & {} if rand < 0.5 \nonumber \\{} & {} if \ abs(A) \ge 1 \nonumber \\{} & {} o_i^{t+1} = x_{best} - n_{best} \times l - o_i^t \times rand() \nonumber \\{} & {} else \nonumber \\{} & {} o_i^{t+1} = x_best \times ge \times rand()\nonumber \\{} & {} elseif \ rand \ge 0.5 \nonumber \\{} & {} de = abs(x_{best} - o_i^t)\nonumber \\{} & {} o_i^{t+1} = de \times exp(l).\times cos(l.2\pi ) + x_{best}\nonumber \\{} & {} end \nonumber \\{} & {} end \end{aligned}$$

where $n_{best}$ is the local best solution, $o_i^t$ is a current solution, *l* is the parameter of PDO and is made self-adaptive by using different mutation operators (discussed in consecutive sections), *A* is a random number.

c) Phase III: For $t_{max}/2$ < iterations $\le$ $t_{max} 3/4$. This phase is controlled by DMA and is used for enhancing the *expl* operation with the advantages of *expt* operation. The DHPN algorithm uses a combination of *expl* and *expt* inspired scout DM phase for position update and is given by

43 $$\begin{aligned} o_i^{t+1}= {\left\{ \begin{array}{ll} o_i - CF \times rand() \times [x_{best} -o_i] \hspace{5pt} if rand()> 0.5 \hspace{5pt} \textit{expl} \\ o_i + CF \times rand() \times [x_{best} -o_i] \hspace{5pt} if rand() > 0.5 \hspace{5pt} \textit{expt} \end{array}\right. } \end{aligned}$$

$rand = [0,1]$, and *CF* is made self-adaptive in nature using different mutation operators. More details on the added parameters is presented in consecutive subsections.

d) Phase IV: iterations > $t_{max} 3/4$. The final phase of iterations is controlled by using HBA. Here, digging phase and honey phase of HBA is used to formulate the basic equations of this phase. The mathematical formulation is given by

44 $$\begin{aligned}{} & {} if \ r < 0.5 \nonumber \\{} & {} o_i^{t+1} = o_i^t + g \times \beta \times I \times o_i^t + g \times rand() \times \alpha \times d_i \times (cos(2 \pi g)) \nonumber \\{} & {} else \nonumber \\{} & {} o_i^{t+1} = o_i^t + F \times \beta \times I \times o_i^t + g \times rand() \times \alpha \times d_i \times (cos(2 \pi g)) \times |[1-cos(2 \pi g)]| \nonumber \\{} & {} end \end{aligned}$$

where $d_i = x_{best}- o_i^t$ is the distance to the best solution, $\beta , and I$ is chosen as 1, $\alpha$ is a random control parameter, and it decreases over iterations to reduce the diversity, *g* is the major parameter of HBA and is optimized using a different set of mutation/*iw* operators. This parameter helps the search agents to change their direction for rigorous *expl*. Apart from that, the major reason for using the digging and honey phase of HBA is because of both *expl* and *expt* search in the basic equations.

#### Breeder phase

This phase is meant to provide better *expt* operation and is evaluated using the current best solutions. Apart from that, $\lambda$ controls the extent of *expt* in the breeder phase. The major reason why this phase is kept as such is because of the inherent properties of a limited number of breeders that remain concentrated around the search space, and hence corresponds to better potential solutions around those sections. In a general scenario, the search agents look for potential solutions that are close to the current/previous best. This helps to exploit the search space efficiently and, hence, improves search capabilities.

### Selection operation

This phase is meant for finding the best solution. Here, the best solutions from the previous and current are compared based on fitness, and the best among both is retained.

45 $$o_{i}^{{t + 1}} = \left\{ {\begin{array}{*{20}l} {o_{i}^{t} } \hfill & {if\;f\left( {o_{i}^{{t + 1}} } \right) < f(o_{i}^{t} )} \hfill \\ {o_{i}^{{t + 1}} } \hfill & {otherwise} \hfill \\ \end{array} } \right.$$

where $f(o_i^t)$ is the fitness of the previous solution and $f(o_i^{t+1})$ signifies the fitness of the current solution.

#### Stagnation phase

To deal with the problems of stagnation, a new phase is incorporated into the proposed DHPN algorithm. This phase uses a combination of SACS^56^ inspired equations for better performance. This phase is activated only if the solution quality is not improving. This helps the algorithm to improve and produce good solutions. Its general equation is given by,

46 $$\begin{aligned}{} & {} o_1=o_i-G_1(Y_1.x_{new}-o_i^t);\hspace{5pt} o_2=o_i-G_2(Y_2.x_{new}-o_i^t);\hspace{5pt} o_3=o_i-G_3(Y_3.x_{new}-o_i^t) \end{aligned}$$

47 $$\begin{aligned}{} & {} x_{i}^{t+1}=\frac{o_1+o_2+o_3}{3} \end{aligned}$$

where $G_1, G_2, G_3 \in A$ and $Y_1,Y_2,Y_3 \in C$ are given by $A=2l.r_1-l;\hspace{5pt} C=2.r_2$. The Eq. (47) is adapted using Cauchy $Cauchy(\delta )$ distributed random parameter and the new equation is

48 $$\begin{aligned} o_i^{t+1}=o_i^t+\alpha \otimes {Cauchy(\delta )}(n_{best}-o_i^t) \end{aligned}$$

The equation for Cauchy distribution is given by

49 $$\begin{aligned} f_{Cauchy(0,g)}(\delta )=\frac{1}{\pi } \frac{g}{\Big ({g^2}+{\delta ^2}\Big )} \end{aligned}$$

The Cauchy distribution function is

50 $$\begin{aligned} y=\frac{1}{2}+\frac{1}{\pi }arctan\left( \frac{\delta }{g}\right) \end{aligned}$$

And $Cauchy(\delta )$ operator is expressed as

51 $$\begin{aligned} \delta =tan\left( \pi \Big (y-\frac{1}{2}\Big )\right) \end{aligned}$$

Here $\delta$ is added because of its fatter tail, and it helps the algorithm to provide better *expl* of the search space. This helps in avoiding local optima and premature convergence. A significant problem to deal with is when to activate this phase. The question is still under consideration, and in our current work, we are activating it if the solution does not change after 10 iterations.

### Parameter settings

The proposed DHPN uses a combination of six new mutation operators/inertia weights (*iw*) for analysing the performance of its six parameters ($\lambda$, *CF*, *P*, *R*, *g* and *l*), and making them adaptive in nature. The mutation operators introduced include, simulated annealing (*sa*), chaotic, exponential decreasing (*exp*), linearly decreasing (*lin*), oscillating, and random *iws*. Note that random weights are added only to see how the algorithm behaves if no adaptive parameter is added. The *iws* are formally given as:

#### Simulated annealing (sa) iw

 This *iw* is meant for providing better *expl* and *expt*^38^ and is mathematically given by

52 $$\begin{aligned} \zeta _k= \zeta _{min}+(\zeta _{max}-\zeta _{min})\times p^{(k-1)} \end{aligned}$$

where $$\zeta _{min}$$, $$\zeta _{max} \in [0, 1]$$ and $$k = 0.95$$.

#### Chaotic iw

This *iw* is meant for improving the global search^41^, and is done by guiding the algorithm away from the local optima.

53 $$\begin{aligned}{} & {} K=4 \times k \times (1-k) \end{aligned}$$

54 $$\begin{aligned}{} & {} \zeta = (\zeta _{max}-\zeta _{min}) \times \frac{t_{max}-t}{t_{max}} + \beta _{min} \times K \end{aligned}$$

where $k \in [0, 1]$, $\beta _{max} = 0.9$, $\beta _{min} = 0.5$, $t_{max}$ is maximum iterations.

#### Exponential decreasing (exp) iw

Exponential *iw* helps in slowly moving the algorithm from *expl* towards *expt*. This helps to achieve better convergence patterns and hence has better *expl* towards the start and *expt* towards the end^42^. It is mathematically given as:

55 $$\begin{aligned} \zeta (t)=\zeta _{min}+(\zeta _{max}-\zeta _{min})exp\left[ -\frac{t}{(\frac{t_{max}}{10})}\right] \end{aligned}$$

where $\zeta _{min}$ and $\zeta _{max}$ lies between [0,1].

#### Linearly decreasing (linear) iw

This *iw* follows a linear pattern for adaptation of parameters, and helps in providing a transitional change from *expl* towards *expt*^38^. In general, this *iw* improve the search for global solutions with better convergence speed. This is given by:

56 $$\begin{aligned} lin = \Bigg (1- \frac{t}{t_{max}}\Big )^{\Big (2 \times \frac{t}{t_{max}}\Big )} \end{aligned}$$

#### Oscillating iw

This *iw* generates periodic waves for balanced *expl* and *expt*, and is mathematically modelled as:

57 $$\begin{aligned}{} & {} \zeta (t)=\frac{\beta _{min}+\zeta _{max}}{2}+\frac{\zeta _{max}-\zeta _{min}}{2}cos\Bigg (\frac{2\pi \,t}{T}\Bigg ) \end{aligned}$$

58 $$\begin{aligned}{} & {} T=\frac{2 \times t_{max}}{3+2k} \end{aligned}$$

where $\zeta _{max} = 0.9$ and $\zeta _{min} = 0.3$, and $k \in [0, 1]$.

**Figure 1:**
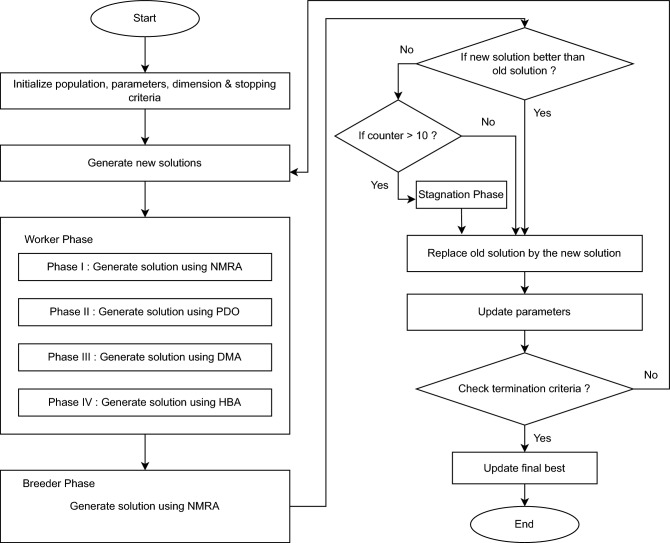
Flowchart of DHPN algorithm.

The flowchart of the proposed algorithm is given in Fig. 1.

The pseudocode of the proposed algorithm is given in Algorithm 1.

**Algorithm 1:**
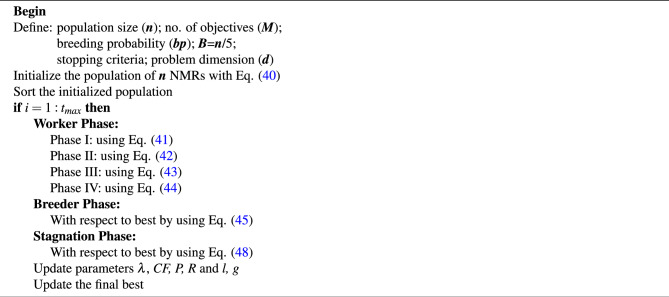
Pseudocode of DHPN algorithm

### Computational complexity

In this section, the computational complexity of the proposed algorithm is analysed with respect to that of the basic algorithm NMRA. If *n* is the size of the population, *d* signifies the dimensionality of the problem and $t_{max}$ represents the maximum number of iterations to find the global optimum, then the computational complexity of the basic NMRA is expressed as O($n.d.t_{max}$)^37^. The complexity analysis is done to examine the operation of an algorithm with worst-case complexities and find the run-time of an algorithm. In the case of a fixed problem dimension, the complexity stands at O(*d*) for an individual population member. However, when the algorithm uses multiple search agents, the complexity increases to O(*n*.*d*), accounting for the population’s size. Given the stochastic nature of the algorithm, evaluation of $t_{max}$ iterations results in an overall complexity of O($n.d.t_{max}$).

In contrast to the original NMRA, the exploration operation in the proposed DHPN algorithm is divided into multiple iterations, but the total number of iterations is kept fixed at $t_{max}$. Thus, there is no change in complexity due to this added adaptation. In terms of addition of stagnation phase, the complexity is given by O(*n*.*d*) and is equal to 1. This is so because the stagnation phase is activated only if the algorithm gets stuck for certain iterations and the new solution is generated only once. Hence, the overall computational complexity of the proposed DHPN algorithm is the same as that of the basic NMRA.

## Results and discussion

This section presents the analysis of DHPN algorithm for different benchmark suites to confirm its efficiency over other algorithms. The section has eight parts, the first subsection consists of CEC 2005 benchmarks. The second and third subsections give the parametric details and analysis of the algorithm under test, respectively. The sensitivity analysis of pop size and dimension size is analysed in the fourth and fifth parts, respectively. The sixth and seventh subsections show the comparative analysis of CEC 2005 and CEC 2019 benchmarks. Finally, the convergence profiles are discussed in the eighth subsection.

The experimental study was performed using a 64-bit Windows 10 operating system, Intel(R) Core(TM) i5-9300H CPU @ 2.40GHz processor, 8.00 GB RAM. Its source code was implemented using MATLAB (R2022b).

### Test suite

It gives details of CEC 2005 benchmarks to analyse the efficiency of the DHPN algorithm. Table 2 gives the description of test functions. These functions can be broadly categorized as unimodal (UM) problems, multimodal (MM) problems, and fixed dimension (FD) problems^48^. The UM problems (*G*1–*G*7) are simplex functions with one global minimal solution and are used to analyse *expl*, whereas MM problems $(G8-G12)$ are used to test the *expl* capability. Such functions have multiple local minimal solutions. On the other hand, as the name suggests, dimension size is fixed for FD functions $(G13-G15)$. These functions analyse the consistency in finding a global solution.

**Table 2:** CEC 2005 Benchmark Dataset.

Function	Dim	Range	Shift position	$f_{min}$
UM functions				
G1(o) = $\sum _{i=1}^{n} o_i^2$	30	$[-100,100]$	$[-30,-30,..,-30]$	0
G2(o) = $\sum _{i=1}^{n} |o_i|+\Pi _{i=1}^n |o_i|$	30	$[-10,10]$	$[-3,-3,..,-3]$	0
G3(o) = $\sum _{i=1}^{n}(\sum _{j-1}^i o_j)^2$	30	$[-100,100]$	$[-3,-3,..,-3]$	0
G4(o) = $mao_i$ $\{$ $|o_i|, 1\le i \le n$ $\}$	30	$[-100,100]$	$[-3,-3,..,-3]$	0
G5(o) = $\sum _{i=1}^{n-1} 100(o_{I+1}-o_i^2)^2+(o_1-1)^2$	30	$[-30,30]$	$[-3,-3,..,-3]$	0
G6(o) = $\sum _{i=1}^{n}([o_i+0.5])^2$	30	$[-10,10]$	$[-3,-3,..,-3]$	0
G7(o) = $\sum _{i=1}^{n} io_i^4 =random [0,1]$	30	$[-1.28,1.28]$	$[-3,-3,..,-3]$	0
MM functions				
G8(o) = $\sum _{i=1}^{n} [o_i^2-10cos(2\pi o_i)+10]$	30	$[-5.12,5.12]$	$[-30,-30,..,-30]$	0
G9(o) = $-20exp(-0.2 \sqrt{\frac{1}{n}}\sum _{i=1}^n o_i^2)-exp(\frac{1}{n}\sum _{i=1}^n cos(2\pi o_i))+20+e$	30	$[-100,100]$	$[-30,-30,..,-30]$	0
G10(o) = $\frac{1}{4000}\sum _{i=1}^N o_i^2 - \Pi _{i=1}^N cos(\frac{o_i}{\sqrt{i}})+1$	30	$[-600,600]$	$[-30,-30,..,-30]$	0
$$\begin{aligned}&\text{G}11(o) =\frac{\pi }{n}10sin(\pi y_1)+\sum _{i=1}^n-1(y_i-1)^2[1+10sin^2(\pi y_{i+1})]\\&{(y_n-1)^2+\sum _{i=1}^u(o_i,10,100,4) y_i=1=\frac{o_i+1}{4}}\end{aligned}$$	30	$[-50,50]$	$[-30,-30,..,-30]$	0
$$\begin{aligned}\text{G}12(o) &= 0.1 ( \sin ^2(3\pi o_1)+\sum _{i=1}^n(o_i-1)^2(1+\sin ^2(3\pi o_i+1)))\\&\quad+0.1((x_n-1)^2[1+\sin ^2(2\pi x_n])+\sum _{i=1}^n u(o_1, 5, 100, 4)\end{aligned}$$	30	$[-50,50]$	$[-30,-30,..,-30]$	0
FD functions				
G13(o)= $[1+(o_1+o_2+1)^2(19-14o_1+3o_1^2-14o_2+6o_1o_2+3o_2^2)]*[30+(2o_1-3o_2)^2*(18-32o_1+12o_1^2+48o_2-36o_1o_2+27o_2^2)]$	2	$[-2,2]$		3
G14(o)= $-\sum _{i=1}^4 c_i \exp (-\sum _{j=1}^3 a_{ij}(o_j-p_{ij})^2)$	3	[1, 3]		$-3.86$
G15(o)= $-\sum _{i=1}^4 c_i \exp (-\sum _{j=1}^6 a_{ij}(o_j-p_{ij})^2)$	6	[0, 1]		$-3.86$

### Preliminary parameter settings

This section describes parameters used for comparative analysis with the proposed DHPN algorithm. For comparison with CEC 2005 benchmarks, success-history based DE (SHADE)^63^, DE with external archive (JADE)^50^, self adaptive DE (SaDE)^64^, opposition and exponential WOA (OEWOA)^65^, fractional-order calculus-based FPA (FA-FPO)^66^, sine cosine crow search (SCCSA)^67^, evolution strategy with covariance adaptation (CMA-ES)^68^, extended GWO (GWO-E)^69^, LSHADE-SPACMA^52^, optimization (DMO) algorithm^39^, HBA^36^ and cuckoo-search (CS) algorithm^70^ are used. Secondly, for CEC 2019 benchmark problems, DHPN is compared with DE, self-adaptive jDE100^71^, particle swarm optimization (PSO)^72^, Young’s double-slit experiment inspired optimizer (YDSE)^73^, naked mole-rat algorithm (NMRA)^37^ and prairie dog optimization (PDO)^40^ algorithm. The parameter details of aforementioned algorithms are based on their corresponding papers and are also provided in Table 3.

**Table 3:** Parameter settings of different algorithms.

Algorithm	Parameters
JADE^50^	$F=0.5$; $1/c=[5,20]$; $CR=0.9$; $p=[0.05,0.20]$
SaDE^50^	$F, CR=$ adaptive
GWO-E^69^	$\mathbf {\alpha }$ = Linearly reducing from 2 to 0
OEWOA^65^	$\mathbf {\alpha }$ = Exponentially reducing function; $b=1$
SCCSA^67^	$r_1, r_2, r_3 = [0,1]$
FA-FPO^66^	$\alpha = [0.1,1]$; $S=adaptive$
SHADE^51^	$P_{best}=0.1$; *ARC* *rate*= 2
LSHADE-SPACMA^51^	*c*=0.8; $P_{best}=0.11$; *ARC* *rate*= 1.4; *FCP*=0.5
YDSE^73^	$\lambda =5\times 10^{-6}$m; $d=5\times 10^{-3}$m; $L=1$m; $I=0.01$m; $\delta =0.38$; $NP=30$
PSO^74^	$W_{max}=0.9$; $C_1$; $W_{min}=0.4$; & $C_2$= 2
DE^74^	*F*=0.5; *CR*=0.9
MPA^75^	$P=0.5$; $\textbf{R}=[0,1]$; $CF=adaptive$
NMRA^37^	$bp=0.5$; $\lambda =[0,1]$
GWO^76^	$\mathbf {\alpha }$ = Linearly reducing [2,0]
WOA^77^	$\mathbf {\alpha }$ = Linearly reducing [2,0]; $b=1$
MFO^78^	$t=[-1,1]$, $b=1$;
HBA^36^	Honey Badger number = 50; $\beta =6$; $C=2$
PDO^40^	$\rho =0.1$; $\varepsilon =2.2204E-16$; $\Delta =0.005$
DHPN	$\lambda$=*chaotic*; $bp=0.05$; $CF=exp$; $\textbf{R}=random$; $P=exp$; $l=exp$; *g*=Linearly decreasing [2,0]

### Sensitivity analysis: parametric study of DHPN algorithm

DHPN algorithm has six important parameters namely $\lambda$, *CF*, *P*, $\textbf{R}$, *l*, and *g*. These parameters are subjected to mutation operators for 3 UM functions (*G*2, *G*6 & *G*7), 1 MM function (*G*11) and 1 FD function (*G*13) of CEC 2005 benchmarks. Results are evaluated as mean values and standard deviation (std) values. In addition, Friedman rank (f-rank)^79^ test values are given in Table 4.

The parameter $\lambda$ is associated with three mutations: simulated annealing *sa* inertia weight (*iw*), *chaotic* and exponential decreasing *exp* *iw*s. Table 4 clearly shows that for $\lambda$, *sa* mutation operator perform the best for *G*2 and *G*13, whereas *exp* mutation operator performs better for *G*6 and *G*7. For *G*11, the parameter $\lambda$ yields the best performance with *chaotic* *iw* in comparison to the other operators. Overall, The parameter *lambda* with *chaotic* mutation operator is the best strategy.

*CF* is checked for three different cases: *linear*, *chaotic* *iw* and *exp* *iw*. Results given in Table 4 depict that the *chaotic* *iw* yields the best solutions for *G*2 and *G*6 for the parameter *CF*. For *G*7, *G*11, and *G*13, *exp* *iw* peforms better compared to the other operators for the parameter *CF*. Overall, the parameter *CF* with *exp* *iw* is found to be the best.

*P* is analysed for a constant *iw*, *chaotic* *iw* and *exp* *iw*. Table 4 shows that the *exp* *iw* outperforms other operators for *G*6, *G*11 and *G*13. For *G*2, the parameter *P* with constant number yields better results. For *G*7, the parameter *P* with *chaotic* *iw* gives the best results. Overall, the parameter *P* with *exp* *iw* outperforms the other operators, and the F-rank statistical test validates the results.

$\textbf{R}$ is analysed for random *iw*, *chaotic* *iw* and *exp* *iw*. Table 4 shows that for the parameter $\textbf{R}$, *chaotic* *iw* performs the best for *G*2 and *G*6, whereas *exp* *iw* is best for *G*7 and *G*13. For *G*11, the random nature of the parameter $\textbf{R}$ outperforms the other operators. Overall, the parameter $\textbf{R}$ with random *iw* is the best.

The last parameters *l* and *g* are analysed using linearly decreasing *linear* *iw*, *oscillating* *iw* and *exp* *iw*. Table 4 that the parameter *l* with *exp* *iw* yields the better results for all problems. Hence, *exp* *iw* is the best strategy for the parameter *l*. Further, the parameter *g* gives the best results with *linear* reduction between [2, 0]. So, here *linear* *iw* of *g* is the best strategy among all the operators.

**Table 4:** Parameter analysis of DHPN algorithm

Parameters of DHPN	Variable Parameters		Functions					Statistical analysis		Best
			*G*2	*G*6	*G*7	*G*11	*G*13	Average f-rank	Overall f-rank	
$\lambda$	$\lambda _{sa}$	Mean	**3.281E−185**	4.441E−01	1.221E−04	1.822E−02	3.000E+00	2	2	$\lambda _{chaotic}$
		std	**0.000E+00**	1.511E−01	2.042E−04	7.271E−03	**2.781E−06**			
		f-rank	1	3	3	2	1			
	$\lambda _{chaotic}$	Mean	1.000E−156	3.781E−01	1.212E−04	**1.581E−02**	3.000E+00	1.8	1	
		std	7.151E−156	1.391E−01	1.200E−04	7.571E−03	1.331E−05			
		f-rank	2	2	2	1	2			
	$\lambda _{exp}$	Mean	1.111E−144	**3.771E−01**	**1.141E−04**	1.911E−02	3.000E+00	2.2	3	
		std	7.911E−144	**1.300E−01**	1.271E−04	2.212E−02	4.262E−05			
		f-rank	3	1	1	3	3			
*CF*	$CF_{linear}$	mean	1.001E−156	3.772E−01	1.211E−04	1.582E−02	3.000E+00	2.6	3	$CF_{exp}$
		std	7.151E−156	1.392E−01	1.201E−04	7.572E−03	1.333E−05			
		f-rank	3	3	2	2	3			
	$CF_{chaotic}$	mean	**4.411E−162**	**3.142E−01**	1.321E−04	1.643E−02	3.000E+00	2	2	
		std	**3.082E−161**	**1.121E−01**	1.352E−04	7.541E−03	4.952E−06			
		f-rank	1	1	3	3	2			
	$CF_{exp}$	Mean	5.242E−159	3.681E−01	**9.452E−05**	**1.511E−02**	3.000E+00	1.4	1	
		std	3.742E−158	1.231E−01	**9.021E−05**	**5.342E−03**	**1.821E−06**			
		f-rank	2	2	1	1	1			
*P*	$P_{0.5}$	mean	**2.421E−159**	3.992E−01	1.441E−04	1.791E−02	3.000E+00	2.6	3	$P_{exp}$
		std	**1.531E−158**	1.552E−01	1.300E−04	1.171E−02	1.622E−05			
		f-rank	1	3	3	3	3			
	$P_{chaotic}$	Mean	2.671E−158	3.771E−01	**9.162E−05**	1.782E−02	3.000E+00	2	2	
		std	1.833E−157	1.171E−01	1.200E−04	1.011E−02	1.440E−05			
		f-rank	3	2	1	2	2			
	$P_{exp}$	Mean	5.242E−159	**3.681E−01**	9.451E−05	**1.511E−02**	3.000E+00	1.4	1	
		std	3.742E−158	**1.231E−01**	9.021E−05	**5.342E−03**	**1.821E−06**			
		f-rank	2	1	2	1	1			
*R*	$R_{random}$	Mean	5.241E−159	3.682E−01	9.452E−05	**1.511E−02**	3.000E+00	1.8	1	$R_{random}$
		std	3.741E−158	1.232E−01	9.021E−05	**5.342E−03**	1.821E−06			
		f-rank	2	2	2	1	2			
	$R_{chaotic}$	Mean	**4.521E−163**	**3.521E−01**	9.922E−05	1.741E−02	3.000E+00	2	2	
		std	**3.141E−162**	1.352E−01	7.573E−05	7.551E−03	2.091E−05			
		f-rank	1	1	3	2	3			
	$R_{exp}$	Mean	2.111E−158	3.800E−01	**9.041E−05**	1.812E−02	3.000E+00	2.2	3	
		std	1.081E−157	1.181E−01	**7.232E−05**	8.451E−03	**1.632E−06**			
		f-rank	3	3	1	3	1			
*l*	$l_{linear}$	Mean	5.241E−159	3.682E−01	9.451E−05	1.511E−02	3.000E+00	2.6	3	$l_{exp}$
		std	3.741E−158	1.231E−01	9.022E−05	5.341E−03	1.822E−06			
		f-rank	3	3	2	3	2			
	$l_{oscillating}$	Mean	7.231E−160	3.672E−01	1.401E−04	1.392E−02	3.000E+00	2.4	2	
		std	5.161E−159	1.400E−01	1.541E−04	6.962E−03	5.421E−06			
		f-rank	2	2	3	2	3			
	$l_{exp}$	Mean	**7.550E−165**	**3.141E−01**	**8.060E−05**	**1.341E−02**	3.000E+00	1	1	
		std	**0.000E+00**	1.271E−01	**6.682E−05**	**5.261E−03**	**1.671E−06**			
		f-rank	1	1	1	1	1			
*g*	$g_{linear}$	Mean	**7.551E−165**	**3.142E−01**	**8.061E−05**	**1.341E−02**	3.000E+00	1	1	$g_{linear}$
		std	**0.000E+00**	**1.271E−01**	**6.681E−05**	**5.261E−03**	**1.672E−06**			
		f-rank	1	1	1	1	1			
	$g_{oscillating}$	Mean	6.97E−161	3.64E−01	1.09E−04	1.65E−02	3.00E+00	2.4	2	
		std	4.98E−160	1.29E−01	1.13E−04	9.99E−03	3.29E−06			
		f-rank	3	2	2	3	2			
	$g_{exp}$	Mean	2.07E−162	3.75E−01	1.40E−04	1.62E−02	3.00E+00	2.6	3	
		std	1.47E−161	1.28E−01	1.29E−04	7.50E−03	2.63E−04			
		f-rank	2	3	3	2	3			

Significant values are in bold.

### Effect of population size

For pop size comparison, DHPN is compared with MFO, GWO, WOA, MPA and NMRA for four pop sizes, including 25, 50, 75 and 100. Total runs and generations are set to 51 and 500, respectively. Here, 7 UM functions (*G*1–Total runs and generations7) and 5 MM functions (*G*8–*G*12) from Table 2 are used for the analysis of various algorithms for different pop sizes as given in Table 5.

*Population size 25:* Here, for *G*3, *G*4, *G*7 and *G*9, DHPN algorithm yields the superior performance for both mean and std values. For *G*1 and *G*2, WOA performs the best, however, DHPN algorithm is quite competitive too. For *G*5, there is a slight variation in the mean values of different algorithms. NMRA is the best for *G*5. For functions *G*6, *G*11 and *G*12, MPA gives the best performance. For *G*8, both MPA and DHPN are capable of yielding the optimum value. For *G*10, the DHPN algorithm, along with MPA and NMRA, is best. Thus, the performance of the DHPN algorithm is best for six problems, MPA is superior for five, and WOA and NMRA for two test problems each. So, DHPN algorithm is found to be the most superior of all the other algorithms for 25 pop size.

*Population size 50:* Here, for *G*1, *G*2, *G*3, *G*4, *G*7 and *G*9, DHPN algorithm outperforms all the other existing algorithms under comparison. For *G*5, NMRA is better. For *G*6, *G*11 and *G*12, MPA is found better. However, for *G*8 and *G*10, MPA, NMRA, and DHPN are capable of giving the optimum values. Hence, the DHPN algorithm gives the best performance for seven, MPA is better for five, and NMRA gives the best result for three. Overall, DHPN is superior for pop size 50.

*Population size 75:* Here DHPN algorithm is better for *G*1, *G*3, *G*7 and *G*9. WOA is found to be the best for *G*2, but the performance of DHPN algorithm is also competitive. NMRA gives the best results for *G*4. For *G*5, NMRA is better among all. For *G*6, *G*11 and *G*12, MPA outperforms others. For *G*8, both MPA and DHPN algorithm yield the optimum value. For *G*10, GWO, MPA, NMRA and DHPN algorithms are better. Hence, the DHPN algorithm is best for six test problems, MPA is best for five, NMRA is best for three, and both WOA and GWO give the best performance for one function only.

*Population size 100:* Here, DHPN outperforms other algorithms for *G*1, *G*3, *G*7 and *G*9. For *G*2, WOA yields better results, but DHPN results are still competitive. For *G*4 and *G*5, NMRA outperforms the other algorithms. For *G*6, *G*11 and *G*12, MPA gives the best results. For *G*8, both MPA and DHPN outperform the other algorithms under comparison. All the algorithms except WOA give the exact optimum value for the *G*10. Therefore, DHPN is superior for six, MPA for five, and NMRA for three, whereas WOA, MFO and GWO perform best for one function each. Therefore, DHPN is best for pop size 100 too.

*Inferences:* Table 5 clearly depicts that the performance of the DHPN algorithm decreases for lesser values of pop size. Further, bigger values do not contribute significantly, but add to the computational burden. This is because, to find a solution for any problem, the required evaluations are a multiple of the total population. With increasing pop size, required function evaluations also increase, thus, resulting in enhanced overall computational complexity. Here, it can be seen that a population of 50 individuals is capable of providing good results without increasing the computational burden of DHPN. Therefore, for simulation results, the pop size is set to 50.

**Table 5:** Experimental results for population size of 25, 50, 75, 100.

Function	Algorithm	Pop Size 25		Pop Size 50		Pop Size 75		Pop Size 100	
		Mean	Std	Mean	Std	Mean	Std	Mean	Std
G1	MFO	1.801E+03	3.831E+03	1.171E+03	3.252E+03	5.891E+02	2.371E+03	9.812E+02	3.000E+03
	GWO	1.381E−25	1.981E−25	3.142E−33	5.321E−33	8.631E−38	1.072E−37	8.772E−41	1.392E−40
	WOA	1.621E−67	1.131E−66	1.111E−83	7.373E−83	6.412E−90	4.372E−89	1.191E−95	7.911E−95
	MPA	2.691E−23	3.481E−23	5.051E−23	4.932E−23	2.761E−23	3.371E−23	2.512E−23	2.552E−23
	NMRA	1.121E−48	7.041E−48	1.122E−86	7.882E−86	5.801E−86	3.952E−85	6.032E−80	4.282E−79
	DHPN	1.351E−66	6.762E−66	0.000E+00	0.000E+00	1.941E−119	9.242E−119	1.071E−118	5.462E−118
G2	MFO	3.211E+01	1.871E+01	3.111E+01	2.000E+01	3.082E+01	2.200E+01	3.100E+01	2.261E+01
	GWO	1.511E−15	1.221E−15	7.111E−20	6.531E−20	2.422E−22	2.011E−22	4.531E−24	4.021E−24
	WOA	5.952E−49	3.582E−42	1.881E−54	5.691E−54	4.062E−56	1.482E−55	3.461E−57	1.211E−56
	MPA	2.241E−13	2.241E−13	3.081E−13	2.981E−13	2.892E−13	2.422E−13	2.262E−13	1.621E−13
	NMRA	6.482E−25	3.782E−24	3.462E−45	1.521E−44	6.911E−44	2.242E−43	1.412E−42	9.111E−42
	DHPN	8.651E−37	6.172E−36	1.411E−188	0.000E+00	3.263E−50	2.332E−49	1.243E−54	8.842E−54
G3	MFO	2.651E+04	1.491E+04	1.891E+04	1.232E+04	1.511E+04	1.193E+04	1.351E+04	9.341E+03
	GWO	8.551E−05	1.952E−04	3.852E−08	6.911E−08	2.201E−10	4.152E−10	1.072E−11	2.401E−11
	WOA	5.101E+04	1.412E+04	2.972E+04	9.331E+03	1.981E+04	6.952E+03	1.622E+04	7.913E+03
	MPA	8.661E−05	1.372E−04	6.711E−05	1.371E−04	1.691E−05	3.782E−05	7.971E−06	1.881E−05
	NMRA	2.341E−47	1.612E−46	2.541E−85	1.323E−84	3.342E−83	1.762E−82	1.482E−83	8.161E−83
	DHPN	7.121E−88	5.081E−87	2.962E−323	0.000E+00	8.200E−154	5.851E−153	2.662E−89	1.901E−88
G4	MFO	6.981E+01	1.000E+01	6.031E+01	9.311E+00	4.631E+01	1.122E+01	4.292E+01	9.341E+00
	GWO	2.323E−06	2.402E−06	2.183E−08	1.742E−08	1.304E−09	1.141E−09	1.951E−10	1.742E−10
	WOA	5.232E+01	2.561E+01	3.723E+01	2.872E+01	3.381E+01	2.911E+01	2.922E+01	2.732E+01
	MPA	3.162E−09	2.132E−09	3.153E−09	1.751E−09	2.214E−09	1.141E−09	1.821E−09	8.542E−10
	NMRA	8.992E−26	3.851E−25	3.502E−45	1.522E−44	6.571E−43	2.731E−42	9.122E−43	3.273E−42
	DHPN	2.341E−36	1.241E−35	1.392E−187	0.000E+00	1.211E−26	4.931E−26	1.952E−27	1.131E−26
G5	MFO	1.581E+06	1.121E+07	1.322E+04	3.112E+04	1.493E+04	3.281E+04	1.164E+04	2.902E+04
	GWO	2.704E+01	7.143E−01	2.672E+01	6.862E−01	2.652E+01	6.581E−01	2.632E+01	6.472E−01
	WOA	2.103E+01	4.631E−01	2.743E+01	4.792E−01	2.701E+01	2.921E−01	2.682E+01	1.902E−01
	MPA	2.542E+01	4.362E−01	2.451E+01	4.371E−01	2.402E+01	4.542E−01	2.353E+01	3.593E−01
	NMRA	2.892E+01	1.922E−02	2.891E+01	2.543E−02	2.891E+01	2.492E−02	2.892E+01	2.711E−02
	DHPN	2.872E+01	1.341E−01	2.832E+01	3.262E−01	2.823E+01	3.771E−01	2.812E+01	4.063E−01
G6	MFO	2.361E+03	5.853E+03	5.922E+02	2.382E+03	1.163E+03	3.233E+03	3.931E+02	1.961E+03
	GWO	9.702E−01	4.022E−01	4.701E−01	2.773E−01	2.822E−01	2.401E−01	1.801E−01	1.982E−01
	WOA	6.182E−01	3.031E−01	8.451E−02	1.202E−01	2.082E−02	3.181E−02	5.601E−03	2.702E−03
	MPA	2.602E−03	1.273E−02	1.431E−08	6.252E−09	7.231E−09	2.733E−09	4.182E−09	1.913E−09
	NMRA	6.843E+00	4.911E−01	6.562E+00	5.902E−01	6.623E+00	6.442E−01	6.531E+00	6.443E−01
	DHPN	6.182E−01	1.992E−01	3.451E−01	1.322E−01	3.182E−01	1.081E−01	2.932E−01	1.011E−01
G7	MFO	6.342E+00	1.102E+01	3.021E+00	7.371E+00	9.182E−01	2.172E+00	1.861E+00	5.823E+00
	GWO	2.302E−03	1.302E−03	1.202E−03	5.082E−04	8.612E−04	4.492E−04	6.122E−04	4.073E−04
	WOA	3.902E−03	4.902E−03	2.301E−03	2.701E−03	1.202E−03	1.302E−03	1.301E−03	1.401E−03
	MPA	1.402E−03	8.011E−04	1.001E−03	4.322E−04	8.401E−04	4.502E−04	8.352E−04	4.013E−04
	NMRA	2.301E−03	2.402E−03	6.942E−04	6.101E−04	5.901E−04	5.522E−04	4.031E−04	3.262E−04
	DHPN	1.772E−04	1.931E−04	8.922E−05	8.192E−05	6.991E−05	6.761E−05	7.001E−05	6.282E−05
G8	MFO	1.691E+02	4.032E+01	1.522E+02	3.041E+01	1.341E+02	3.362E+01	1.432E+02	4.093E+01
	GWO	5.051E+00	5.842E+00	1.642E+00	3.063E+00	2.281E+00	3.842E+00	7.122E−01	1.931E+00
	WOA	2.231E−15	1.592E−14	1.111E−15	7.951E−15	1.111E−15	7.951E−15	4.452E−15	1.912E−14
	MPA	0.000E+00	0.000E+00	0.000E+00	0.000E+00	0.000E+00	0.000E+00	0.000E+00	0.000E+00
	NMRA	2.431E+00	1.741E+01	0.000E+00	0.000E+00	2.361E+00	1.681E+01	6.422E+00	3.211E+01
	DHPN	0.000E+00	0.000E+00	0.000E+00	0.000E+00	0.000E+00	0.000E+00	0.000E+00	0.000E+00
G9	MFO	1.581E+01	5.951E+00	1.162E+01	8.602E+00	1.022E+01	9.371E+00	9.541E+00	8.812E+00
	GWO	2.042E−13	5.471E−13	4.261E−14	3.322E−14	3.182E−14	4.331E−15	2.822E−14	2.862E−15
	WOA	4.992E−15	2.961E−15	4.441E−15	2.242E−15	4.722E−15	2.541E−15	4.092E−15	2.772E−15
	MPA	1.712E−12	1.133E−12	1.441E−12	8.942E−13	1.222E−12	7.142E−13	1.261E−12	6.000E−13
	NMRA	8.888E−16	0.000E+00	8.888E−16	0.000E+00	8.888E−16	0.000E+00	8.888E−16	0.000E+00
	DHPN	4.444E−16	0.000E+00	4.444E−16	0.000E+00	4.444E−16	0.000E+00	4.444E−16	0.000E+00
G10	MFO	3.332E−02	7.541E−02	5.701E−03	2.852E−02	2.901E−03	2.041E−02	0.000E+00	0.000E+00
	GWO	8.602E−03	3.461E−02	8.601E−03	3.462E−02	0.000E+00	0.000E+00	0.000E+00	0.000E+00
	WOA	3.432E−02	6.241E−02	4.281E−02	6.702E−02	1.712E−02	4.741E−02	1.431E−02	4.372E−02
	MPA	0.000E+00	0.000E+00	0.000E+00	0.000E+00	0.000E+00	0.000E+00	0.000E+00	0.000E+00
	NMRA	0.000E+00	0.000E+00	0.000E+00	0.000E+00	0.000E+00	0.000E+00	0.000E+00	0.000E+00
	DHPN	0.000E+00	0.000E+00	0.000E+00	0.000E+00	0.000E+00	0.000E+00	0.000E+00	0.000E+00
G11	MFO	7.201E+04	5.051E+05	5.322E+00	6.642E+00	2.471E+00	1.521E+00	2.512E+00	1.822E+00
	GWO	5.843E−02	3.223E−02	2.592E−02	1.312E−02	1.791E−02	1.161E−02	1.552E−02	8.702E−03
	WOA	3.363E−02	2.131E−02	7.401E−03	6.302E−03	2.602E−03	4.303E−03	1.901E−03	7.202E−03
	MPA	2.432E−04	8.851E−04	1.391E−09	6.502E−10	6.192E−10	3.341E−10	4.213E−10	1.762E−10
	NMRA	1.032E+00	2.541E−01	1.093E+00	2.563E−01	1.042E+00	2.492E−01	1.103E+00	2.313E−01
	DHPN	2.651E−02	1.651E−02	1.642E−02	7.611E−03	1.622E−02	9.902E−03	1.143E−02	5.101E−03
G12	MFO	8.041E+06	5.741E+07	8.022E+00	7.712E+00	4.843E+00	6.763E+00	3.752E+00	3.082E+00
	GWO	7.392E−01	2.363E−01	3.343E−01	2.021E−01	2.372E−01	1.862E−01	1.872E−01	1.403E−01
	WOA	7.443E−01	2.732E−01	2.253E−01	1.601E−01	7.283E−02	7.062E−02	4.321E−02	7.213E−02
	MPA	3.000E−02	3.573E−02	6.532E−04	2.602E−03	8.613E−04	3.000E−03	4.592E−04	2.202E−03
	NMRA	2.083E+00	3.653E−01	2.972E+01	1.382E−01	2.983E+00	1.101E−02	3.023E+00	1.182E−01
	DHPN	3.143E−01	1.511E−01	2.361E−01	1.051E−01	2.151E−01	8.382E−02	1.602E−01	5.661E−02

### Effect of dimension size

This subsection describes the dimension size effect on DHPN algorithm with MFO, GWO, MPA, WOA, and NMRA. Here, 7 UM functions (*G*1–*G*7) and 5 MM functions (*G*8–*G*12) from Table 2 are used for analysis. Total runs and iterations is 51 and 500, respectively. To study the effect of dimension size, four dimension sizes such as 10, 30, 50 and 100 are chosen. The results obtained for different dimension sizes are given in Table 6.

*Dimension size 10:* Here, DHPN algorithm is better for *G*1, *G*4, *G*7, *G*2, *G*3, *G*8 and *G*9. For *G*5, NMRA gives superior results in terms of std values. For *G*6, *G*11, and *G*12, MPA outperforms others. GWO, MPA, NMRA and the proposed DHPN algorithm are capable of giving the exact global minima for *G*10. Overall, the proposed DHPN algorithm gives the best solutions for eight, MPA for five, NMRA for two, and GWO for one function only.

*Dimension size 30:* Here, DHPN algorithm outperforms others for *G*1, *G*2, *G*3, *G*4, *G*7 and *G*9. For *G*5, NMRA performs betters; for *G*6, *G*11 and *G*12, MPA yields the better solutions. MPA, NMRA and the proposed DHPN algorithm give the exact global minima for *G*8 and *G*10. Overall, DHPN performs better for eight, MPA for five and NMRA for two test problems.

*Dimension size 50:* Here, DHPN is the best one for *G*1, *G*2, *G*3, *G*4, *G*7 and *G*9. Further, for *G*5, NMRA gives the best for std values. MPA gives better performance for *G*6, *G*11 and *G*12. For *G*8 and *G*10, MPA, NMRA, and the proposed DHPN algorithm give improved performance. Here, the DHPN algorithm is better for eight, MPA is better for five, and NMRA is better for two functions.

*Dimension size 100:* Here, the proposed DHPN algorithm yields the best results for *G*1, *G*2, *G*3, *G*4, *G*6, *G*7 and *G*9 for both mean as well as std values. For *G*5, NMRA is found to be the best. For *G*8, WOA, NMRA, MPA, and DHPN are capable of yielding the optimum value. For *G*10, all the algorithms except MFO and WOA give the desired global optimum value. For *G*11 and *G*12, WOA and the proposed DHPN algorithm give better results, respectively. Hence, the proposed DHPN is better for ten problems, NMRA for three, WOA and MPA for two, and GWO for one function only. In the nutshell, the proposed DHPN is best for 100 dimension size.

*Inference:* Simulation results clearly indicate that the DHPN algorithm is the best overall strategy for all the dimension sizes. For the rest of the simulation results, the value of dimension size is set to 30.

**Table 6:** Experimental results for dimension size of 10, 30, 50, 100.

Function	Algorithm	Dim Size 10		Dim Size 30		Dim Size 50		Dim Size 100	
		Mean	Std	Mean	Std	Mean	Std	Mean	Std
G1	MFO	5.272E−14	8.781E−14	1.171E+03	3.252E+03	5.722E+03	7.253E+03	5.393E+04	1.343E+04
	GWO	6.912E−69	4.002E−68	3.141E−33	5.321E−32	9.662E−24	1.002E−23	5.283E−15	4.791E−15
	WOA	4.342E−88	2.751E−87	1.111E−83	7.372E−83	5.862E−83	3.333E−82	2.663E−82	1.193E−81
	MPA	8.042E−31	1.361E−30	5.052E−23	4.932E−23	3.651E−21	3.041E−21	3.712E−19	3.622E−19
	NMRA	3.000E−81	2.141E−80	1.121E−86	7.882E−86	4.332E−88	2.372E−87	6.663E−86	3.351E−85
	DHPN	0.000E+00	0.000E+00	0.000E+00	0.000E+00	0.000E+00	0.000E+00	0.000E+00	0.000E+00
G2	MFO	7.841E−01	2.711E+00	3.111E+01	2.000E+01	6.602E+01	3.000E+01	2.351E+02	4.191E+01
	GWO	4.071E−40	6.811E−40	7.111E−20	6.532E−20	1.372E−14	7.652E−15	1.553E−09	5.47§E−10
	WOA	5.241E−55	2.061E−54	1.882E−54	5.691E−54	2.553E−52	1.592E−51	4.212E−54	9.561E−54
	MPA	7.862E−18	8.912E−18	3.082E−13	2.982E−13	3.042E−12	2.633E−12	2.441E−11	2.722E−11
	NMRA	1.833E−44	9.361E−44	3.462E−45	1.522E−44	4.952E−44	3.373E−43	8.733E−45	2.772E−44
	DHPN	0.000E+00	0.000E+00	1.411E−188	0.000E+00	5.511E−188	0.000E+00	1.341E−183	0.000E+00
G3	MFO	9.801E+01	7.000E+02	1.892E+04	1.231E+04	5.821E+04	2.072E+04	2.232E+05	5.701E+04
	GWO	1.271E−31	7.381E−31	3.852E−08	6.912E−08	3.401E−02	1.941E−01	1.461E+02	2.542E+02
	WOA	4.181E+01	8.751E+01	2.972E+04	9.332E+03	1.551E+05	2.523E+04	9.002E+05	1.853E+05
	MPA	9.371E−15	1.552E−14	6.712E−05	1.372E−04	2.431E−02	4.173E−02	5.582E+00	6.674E+00
	NMRA	6.401E−89	2.871E−88	2.542E−85	1.322E−84	9.701E−87	6.853E−86	1.992E−87	9.354E−87
	DHPN	0.000E+00	0.000E+00	2.961E−323	0.000E+00	8.531E−298	0.000E+00	8.972E−301	0.000E+00
G4	MFO	6.362E−02	3.062E−01	6.031E+01	9.311E+00	7.922E+01	5.762E+00	9.141E+01	2.411E+00
	GWO	1.712E−22	3.091E−22	2.181E−08	1.742E−08	3.582E−05	2.883E−05	1.983E−01	2.332E−01
	WOA	1.553E+00	4.461E+00	3.721E+01	2.872E+01	6.312E+01	2.953E+01	7.142E+01	2.812E+01
	MPA	6.702E−13	4.562E−13	3.151E−09	1.751E−09	2.582E−08	1.062E−08	2.763E−07	1.173E−07
	NMRA	4.341E−45	2.662E−44	3.502E−45	1.523E−44	9.453E−42	6.752E−41	6.472E−45	3.161E−44
	DHPN	5.222E−185	0.000E+00	1.391E−187	0.000E+00	7.651E−181	0.000E+00	8.353E−185	0.000E+00
G5	MFO	5.731E+03	2.131E+04	1.322E+04	3.111E+04	3.523E+06	1.562E+07	1.232E+08	5.663E+07
	GWO	6.352E+00	7.064E−01	2.671E+01	6.863E−01	4.722E+01	8.322E−01	9.743E+01	8.542E−01
	WOA	6.292E+00	4.851E−01	2.743E+01	4.792E−01	4.772E+01	4.253E−01	9.781E+01	2.974E−01
	MPA	1.011E+00	3.052E−01	2.452E+01	4.371E−01	4.533E+01	3.502E−01	9.624E+01	7.833E−01
	NMRA	8.97E1+00	2.042E−02	2.892E+01	2.541E−02	4.893E+01	2.002E−02	9.893E+01	1.873E−02
	DHPN	7.622E+00	4.432E−01	2.831E+01	3.263E−01	4.842E+01	1.700E−01	9.800E+01	9.171E−02
G6	MFO	1.500E−13	4.251E−13	5.921E+02	2.382E+03	7.402E+03	8.693E+03	5.443E+04	1.404E+04
	GWO	2.571E−06	9.732E−07	4.702E−01	2.773E−01	1.831E+00	6.352E−01	8.093E+00	9.963E−01
	WOA	1.252E−04	9.456E−05	8.453E−02	1.201E−01	3.574E−01	1.562E−01	1.892E+00	6.073E−01
	MPA	7.021E−12	5.543E−12	1.432E−08	6.251E−09	2.263E−02	5.262E−02	2.164E+00	6.131E−01
	NMRA	1.292E+00	7.472E−01	6.561E+00	5.903E−01	1.173E+01	4.844E−01	2.431E+01	4.664E−01
	DHPN	4.572E−02	2.951E−02	3.452E−01	1.322E−01	7.713E−01	1.822E−01	1.761E+00	5.083E−01
G7	MFO	5.501E−03	3.000E−03	3.022E+00	7.371E+00	1.921E+01	2.532E+01	2.452E+02	1.362E+02
	GWO	4.682E−04	3.231E−04	1.203E−03	5.082E−04	2.202E−03	9.951E−04	4.703E−03	1.502E−03
	WOA	1.602E−03	2.101E−03	2.303E−03	2.702E−03	2.803E−03	3.403E−03	2.803E−03	3.404E−03
	MPA	5.923E−04	3.661E−04	1.000E−03	4.322E−04	1.100E−03	3.722E−04	1.401E−03	6.731E−04
	NMRA	8.181E−04	7.682E−04	6.942E−04	6.103E−04	6.443E−04	6.522E−04	6.652E−04	5.163E−04
	DHPN	1.021E−04	1.021E−04	8.922E−05	8.193E−05	8.231E−05	7.552E−05	9.243E−05	7.972E−05
G8	MFO	1.981E+01	1.141E+01	1.522E+02	3.042E+01	2.921E+02	5.232E+01	8.192E+02	7.313E+01
	GWO	2.902E−01	1.071E+00	1.641E+00	3.062E+00	3.632E+00	4.623E+00	7.332E+00	5.932E+00
	WOA	7.491E−01	5.353E+00	1.112E−15	7.952E−15	0.000E+00	0.000E+00	0.000E+00	0.000E+00
	MPA	3.161E−11	2.261E−10	0.000E+00	0.000E+00	0.000E+00	0.000E+00	0.000E+00	0.000E+00
	NMRA	1.381E+00	5.721E+00	0.000E+00	0.000E+00	0.000E+00	0.000E+00	0.000E+00	0.000E+00
	DHPN	0.000E+00	0.000E+00	0.000E+00	0.000E+00	0.000E+00	0.000E+00	0.000E+00	0.000E+00
G9	MFO	6.881E−08	6.012E−08	1.162E+01	8.601E+00	1.902E+01	1.692E+00	1.982E+01	1.263E−01
	GWO	5.831E−15	1.751E−15	4.262E−14	3.322E−15	5.323E−13	1.333E−13	7.092E−09	2.742E−09
	WOA	4.021E−15	2.311E−15	4.442E−15	2.242E−15	4.091E−15	2.483E−15	5.062E−15	2.212E−15
	MPA	4.511E−15	4.972E−15	1.442E−12	8.941E−13	9.613E−12	4.792E−12	6.302E−11	2.973E−11
	NMRA	8.888E−16	0.000E+00	8.888E−16	0.000E+00	8.888E−16	0.000E+00	8.888E−16	0.000E+00
	DHPN	4.444E−16	0.000E+00	4.444E−16	0.000E+00	4.444E−16	0.000E+00	4.444E−16	0.000E+00
G10	MFO	1.141E−02	3.951E−02	5.702E−03	2.852E−02	2.903E−03	2.043E−02	1.142E−02	3.951E−02
	GWO	0.000E+00	0.000E+00	8.602E−03	3.462E−02	5.701E−03	2.851E−02	0.000E+00	0.000E+00
	WOA	3.431E−02	6.241E−02	4.282E−02	6.702E−02	2.281E−02	5.351E−02	3.142E−02	6.052E−02
	MPA	0.000E+00	0.000E+00	0.000E+00	0.000E+00	0.000E+00	0.000E+00	0.000E+00	0.000E+00
	NMRA	0.000E+00	0.000E+00	0.000E+00	0.000E+00	0.000E+00	0.000E+00	0.000E+00	0.000E+00
	DHPN	0.000E+00	0.000E+00	0.000E+00	0.000E+00	0.000E+00	0.000E+00	0.000E+00	0.000E+00
G11	MFO	1.701E−01	4.622E−01	5.322E+00	6.643E+00	1.531E+07	6.072E+07	2.292E+08	1.701E+08
	GWO	1.801E−03	5.602E−03	2.592E−02	1.311E−02	8.641E−02	6.702E−02	2.102E−01	5.813E−02
	WOA	2.402E−03	6.000E−03	7.401E−03	6.301E−03	1.142E−02	5.701E−03	1.811E−02	1.192E−02
	MPA	3.442E−12	3.000E−12	1.391E−09	6.501E−10	1.302E−03	1.402E−03	2.632E−02	7.201E−03
	NMRA	8.241E−01	4.011E−01	1.092E+00	2.562E−01	1.161E+00	1.442E−01	1.202E+00	7.581E−02
	DHPN	1.131E−02	8.801E−03	1.641E−02	7.611E−03	1.791E−02	8.101E−03	1.802E−02	5.402E−03
G12	MFO	2.601E−03	4.701E−03	8.022E+00	7.711E+00	1.682E+07	8.022E+07	4.621E+08	2.803E+08
	GWO	1.901E−03	1.361E−02	3.342E−01	2.022E−01	1.582E+00	3.383E−01	5.991E+00	4.462E−01
	WOA	5.301E−03	8.801E−03	2.252E−01	1.602E−01	4.911E−01	2.332E−01	1.612E+00	6.873E−01
	MPA	1.751E−11	1.791E−11	6.532E−04	2.602E−03	8.581E−02	7.242E−02	5.622E+00	2.812E+00
	NMRA	8.633E−01	1.761E−01	2.973E+00	1.382E−01	4.992E+00	2.502E−03	9.993E+00	2.003E−03
	DHPN	5.433E−02	3.863E−02	2.361E−01	1.052E−01	4.672E−01	1.463E−01	9.511E−01	2.843E−01

### Comparison for CEC 2005 benchmarks

Here, DHPN algorithm is evaluated against the existing optimization algorithms such as JADE, OEWOA, FA-FPO, SCCSA, GWO-E, SaDE, HBA, LSHADE-SPACMA, CMA-ES, SHADE, DMO, and CS for CEC 2005 benchmark functions^48^. The parameters of the aforementioned algorithms are shown in Table 3. Here, a dimension size of 30 and a number of runs of 51 is taken.

#### Experimental studies

Comparative results in Table 7 clearly depict that for *G*1, *G*2, *G*3, *G*4, *G*7 and *G*9, only the proposed DHPN algorithm yields better solutions while the other algorithms may result in sub-optimal solutions. For *G*5, *G*11 and *G*12 JADE is better; and for *G*6, CMS-ES outperforms others. For *G*8, GWO-E, OEWOA, FA-FPO, HBA and DHPN algorithms are capable of providing optimum solution. Further, for *G*10, GWO-E, FA-FPO, DMO, HBA and DHPN algorithms yield optimal solution. For *G*13, little changes in the mean can be seen and on comparing the std values, DMO gives the best results. Similarly, for *G*14, a slight variation in the mean values of different algorithms is observed. So, on the basis of std, JADE is the best. Further, for *G*15, OEWOA is found to be the best. Hence, among fifteen functions, the proposed DHPN algorithm is best for eight, JADE for four, OEWOA, GWO-E, FA-FPO, DMO and HBA for two, whereas CMA-ES for one function only. So, overall, the proposed DHPN algorithm outperforms others.

#### Statistical testing

To validate the simulation results, two non-parametric tests namely Wilcoxon’s p-rank^80^ and Friedman f-rank tests^79^ are employed. The p-rank is used in assigning the p-values to two algorithms under test and is given in Table 7 for every problem as *win*(*w*)/*loss*(*l*)/*tie*(*t*). Here $win(w) = '+'$ situation, test algorithm yields better performance than the proposed; $loss(l) = '-'$ situation arises when the performance of the test algorithm is inferior to the proposed; and lastly, the *tie*(*t*) scenario arises when both the considered algorithms are either significant or irrelevant and hence, assigned ”=” is assigned to it. So, from *w*/*l*/*t* in Table 7, we find that the DHPN algorithm is better than existing algorithms. This test is also applied to validate the superiority of the proposed DHPN algorithm. In this test, each algorithm is given a unique rank and is shown in Table 7. It has been found that the DHPN algorithm is comparatively better and has the $1^{st}$ rank among all others.

**Table 7:** Simulation results for CEC 2005 benchmark functions

Function		JADE ^50^	SaDE ^50^	GWO-E ^69^	OEWOA ^65^	SCCSA ^67^	FA-FPO ^66^	CMA-ES ^51^	SHADE ^51^	LSHADE- SPACMA^51^	DMO ^39^	HBA ^36^	CS ^70^	DHPN
*G*1	mean	1.802E−60	4.501E−20	3.922E−67	7.752E−176	9.221E−69	1.511E−184	1.422E−18	1.421E−09	2.232E−01	5.721E+00	1.111E−146	1.552E+01	**0.000E+00**
	std	8.401E−60	6.901E−20	1.111E−66	**0.000E+00**	3.811E−68	**0.000E+00**	3.131E−18	3.092E−09	1.482E−01	2.022E+00	6.262E−146	4.451E+00	**0.000E+00**
	p-rank	−	−	−	−	−	−	−	−	−	−	−	−	
	f-rank	7	8	6	3	5	2	9	10	11	12	4	13	1
*G*2	mean	1.801E−25	1.902E−14	4.311E−36	1.862E−115	8.251E−41	5.042E−93	2.981E−07	8.702E−03	2.111E+01	2.522E+01	3.302E−78	2.000E+01	**1.411E−188**
	std	8.802E−25	1.053E−14	6.572E−36	1.321E−114	4.192E−40	3.472E−93	1.781E+00	2.132E−02	9.573E+00	2.252E+01	8.432E−78	4.523E+00	**0.000E+00**
	p-rank	−	−	−	−	−	−	−	−	−	−	−	−	
	f-rank	7	8	6	2	5	3	9	10	12	13	4	11	1
*G*3	mean	5.701E−61	9.000E−37	3.752E−37	2.872E+04	4.311E−13	1.232E−183	1.592E−05	1.543E+01	8.871E+01	5.942E+04	1.533E−105	3.101E+03	**2.962E−323**
	std	2.701E−60	5.432E−36	1.362E−36	1.021E+04	2.832E−30	**0.000E+00**	2.212E−05	9.943E+00	4.721E+01	1.032E+04	8.733E−105	6.334E+02	**0.000E+00**
	p-rank	−	−	−	−	−	−	−	−	−	−	−	−	
	f-rank	4	6	5	12	7	2	8	9	10	13	3	11	1
*G*4	mean	8.202E−24	7.400E−11	2.391E−25	1.062E+01	2.151E−17	9.973E−93	2.012E−06	9.793E−01	2.111E+00	5.543E+01	4.422E−62	1.124E+01	**1.392E−187**
	std	4.000E−23	1.822E−10	6.802E−25	2.223E+01	1.063E−16	7.311E−93	1.251E−06	7.994E−01	4.922E−01	5.213E+00	1.201E−61	1.223E+00	**0.000E+00**
	p-rank	−	−	−	−	−	−	−	−	−	−	−	−	
	f-rank	5	7	4	11	6	2	8	9	10	13	3	12	1
*G*5	mean	**8.000E−02**	2.100E+01	2.653E+01	2.853E+01	5.902E+00	2.891E+01	3.672E+01	2.441E+01	2.883E+01	1.573E+05	2.301E+01	8.111E+02	2.831E+01
	std	5.601E−01	7.80E+00	5.191E−01	2.224E−01	9.132E−01	**1.723E−02**	3.342E+01	1.122E+01	8.243E−01	8.262E+04	7.712E−01	3.293E+02	3.262E−01
	p-rank	+	$+$	$+$	−	+	−	−	$+$	−	−	+	−	
	f-rank	1	3	6	8	2	10	11	5	9	13	4	12	7
*G*6	mean	2.901E+00	9.302E+02	2.652E+01	1.622E+00	4.143E−08	5.881E+00	**6.832E−19**	5.311E−10	2.483E−01	5.602E+00	4.021E−05	1.632E+01	3.452E−01
	std	1.202E+00	1.801E+02	5.192E−01	6.931E−01	5.223E−08	5.862E−01	**6.711E−19**	6.352E−10	1.131E−01	1.963E+00	1.883E−04	5.244E+00	1.323E−01
	p-rank	−	−	−	−	+	−	+	+	+	−	+	−	
	f-rank	8	13	12	7	3	10	1	2	5	9	4	11	6
*G*7	mean	6.401E−04	4.802E−03	9.901E−04	1.373E−03	1.332E−03	1.134E−04	2.752E−02	2.353E−02	4.701E−03	2.443E−01	2.893E−04	7.693E−02	**8.924E−05**
	std	2.502E−04	1.201E−03	8.372E−04	2.854E−03	1.723E−03	8.944E−04	7.901E−03	8.802E−03	1.902E−03	7.024E−02	1.982E−04	2.453E−02	**8.192E−05**
	p-rank	−	−	−	−	−	−	−	−	−	−	−	−	
	f-rank	4	9	5	7	6	2	11	10	8	13	3	12	1
*G*8	mean	1.000E−04	1.202E−03	**0.000E+00**	**0.000E+00**	5.461E+00	**0.000E+00**	2.531E+01	8.533E+00	6.752E+01	2.300E+02	**0.000E+00**	1.082E+02	**0.000E+00**
	std	6.000E−05	6.501E−04	**0.000E+00**	**0.000E+00**	5.622E+00	**0.000E+00**	8.552E+00	2.194E+00	1.000E+01	1.600E+01	**0.000E+00**	1.411E+01	**0.000E+00**
	p-rank	−	−	$=$	$=$	−	$=$	−	−	−	−	$=$	−	
	f-rank	6	7	1	1	8	1	10	9	11	13	1	12	1
*G*9	mean	8.200E−10	2.702E−03	5.581E−15	3.021E−15	8.888E−16	8.888E−16	1.551E+01	3.951E−01	3.933E−02	3.102E+00	3.912E−01	9.083E+00	**4.444E−16**
	std	6.900E−10	5.101E−04	1.672E−15	2.272E−15	9.361E−32	**0.000E+00**	7.921E+00	5.862E−01	1.511E−02	4.663E−01	2.792E+00	2.201E+00	**0.000E+00**
	p-rank	−	−	−	−	−	−	−	−	−	−	−	−	
	f-rank	6	7	5	4	3	2	13	10	8	11	9	12	1
*G*10	mean	9.900E−08	7.802E−04	**0.000E+00**	1.421E−02	3.332E−02	**0.000E+00**	5.761E−15	4.803E−03	8.941E−01	**0.000E+00**	**0.000E+00**	2.901E−15	**0.00E+00**
	std	6.000E−07	1.201E−03	**0.000E+00**	1.000E−01	4.563E−02	**0.000E+00**	6.182E−15	7.704E−03	1.073E−01	**0.000E+00**	**0.000E+00**	5.251E−15	**0.000E+00**
	p-rank	−	−	$=$	−	−	$=$	−	−	−	$=$	$=$	−	
	f-rank	8	9	1	11	12	1	7	10	13	1	1	6	1
*G*11	mean	**4.600E−17**	1.901E−05	1.983E−02	1.061E−01	1.342E−02	8.321E−01	2.871E−16	3.462E−02	8.181E−04	7.183E+04	3.151E−07	3.382E+00	1.644E−02
	std	**1.900E−16**	9.202E−06	1.012E−02	4.973E−02	1.603E−02	1.783E−01	5.643E−16	8.754E−02	1.000E−03	9.622E+04	3.473E−07	7.253E−01	7.613E−03
	p-rank	+	+	−	−	$+$	−	+	−	+	−	+	−	
	f-rank	1	4	8	10	6	11	2	9	5	13	3	12	7
*G*12	mean	**2.000E−16**	6.100E−05	2.501E−01	1.033E+00	2.011E−02	2.944E+00	3.663E−04	7.321E−04	1.021E−02	2.422E+05	1.613E−01	8.023E+00	2.363E−01
	std	**6.500E−16**	2.000E−05	1.632E−01	3.614E−01	7.233E−02	1.592E−01	2.000E−03	2.802E−03	1.033E−02	2.203E+05	1.342E−01	1.662E+00	1.052E−01
	p-rank	+	+	−	−	+	−	+	+	+	−	+	−	
	f-rank	1	2	9	10	6	11	3	4	5	13	7	12	8
*G*13	mean	3.000E+00	3.000E+00	3.000E+00	3.000E+00	3.000E+00	3.000E+00	8.400E+00	3.000E+00	3.000E+00	3.000E+00	3.000E+00	3.000E+00	3.000E+00
	std	1.100E−15	3.000E−15	1.252E−05	4.961E−04	8.932E−05	3.131E−09	2.052E+01	1.873E−15	1.252E−15	**5.441E−16**	1.393E−15	1.532E−15	1.053E−05
	p-rank	+	+	−	−	−	+	−	+	+	+	+	+	
	f-rank	2	7	10	12	11	8	13	6	3	1	4	5	9
*G*14	mean	− 3.861E+00	− 3.861E+00	− 3.862E+00	− 3.863E+00	− 3.863E+00	− 3.013E−01	− 3.863E+00	− 3.863E+00	− 3.863E+00	− 3.862E+00	− 3.862E+00	− 3.863E+00	− 3.863E+00
	std	**0.000E+00**	3.100E−15	4.161E−06	2.923E−04	9.292E−06	2.252E−16	2.701E−15	2.692E−15	2.703E−15	1.353E−15	1.903E−03	1.102E−15	7.053E−04
	p-rank	+	+	+	+	+	+	+	+	+	+	−	+	
	f-rank	1	8	9	11	10	2	6	5	6	4	13	3	12
*G*15	mean	− 3.311E+00	− 3.311E+00	− 3.262E+00	**− 3.243E+00**	− 3.262E+00	− 3.293E+00	− 3.292E+00	− 3.271E+00	− 3.283E+00	− 3.324E+00	**− 3.242E+00**	− 3.323E+00	− 3.253E+00
	std	3.602E−02	2.802E−02	7.504E−02	8.182E−02	6.000E−02	1.970E−02	5.351E−02	5.992E−02	5.703E−02	**7.264E−15**	6.892E−02	1.583E−07	7.054E−02
	p-rank	−	−	−	+	−	−	−	−	−	−	+	−	
	f-rank	11	10	5	1	4	8	9	6	7	12	2	13	3
w/l/t		5/10/0	5/10/0	2/11/2	2/12/1	5/10/0	2/11/2	4/11/0	5/10/0	5/10/0	2/12/1	6/7/2	2/13/0	NA
Overall f-rank value		72	108	92	110	94	75	120	114	123	154	65	157	60
Overall f-rank		3	7	5	8	6	4	10	9	11	12	2	13	1

Significant values are in bold.

### Comparison on CEC 2019 benchmark functions

As a further extension, the competitiveness of DHPN algorithm is proven by testing it on ten CEC 2019 benchmarks^71^ of the 100-Digit Challenge^81^. Table 8 list the names, dimensions, search ranges and optimal values of CEC 2019 benchmark functions $(G16-G25)$. For computing simulation results on CEC 2019 functions, the values of pop size, runs and maximum iterations are taken as 50, 51 and 500, respectively. Further, existing algorithms jDE100, DE, PSO, YDSE, NMRA and PDO are compared with the proposed DHPN algorithm as mean and std values in Table 9.

**Table 8:** CEC 2019 Benchmark Functions.

No.	Function	Dim	Range	$f_{min}$
G16	Storn’s Chebyshev Polynomial Fitting Problem	9	$[-8192,8192]$	1
G17	Inverse Hilbert Matrix Problem	16	$[-16384,16384]$	1
G18	Lennard-Jones Minimum Energy Cluster Problem	18	$[-4,4]$	1
G19	Rastrigin’s Problem	10	$[-100,100]$	1
G20	Griewangk’s Problem	10	$[-100,100]$	1
G21	Weierstrass Problem	10	$[-100,100]$	1
G22	Modified Schwefel’s Problem	10	$[-100,100]$	1
G23	Expanded Schaffer’s G6 Problem	10	$[-100,100]$	1
G24	Happy Cat Problem	10	$[-100,100]$	1
G25	Ackley’s Problem	10	$[-100,100]$	1

Table 9 shows that the proposed DHPN is best for functions *G*21, *G*22 and *G*25; for *G*16 and *G*18, PDO is better; and for *G*17, YDSE yields the best performance. PSO yields best results for *G*19, *G*20, *G*23 and *G*24. Here also, Wilcoxon’s p-rank and f-rank tests are done to test the significance of the DHPN algorithm. From Table 9, we find that the DHPN algorithm has 10 wins and no loss against jDE100. As compared to both DE and PDO, DHPN has 8 wins and 2 losses with no draw. Similarly, DHPN has a total of 6 wins and 4 losses with no draw against PSO and YDSE. Moreover, according to the f-rank test, DHPN stands at $1^{st}$ among all comparative algorithms. Thus, DHPN is deduced to be the best overall strategy for the CEC 2019 benchmark suite.

**Table 9:** Statistical results for 100-digit challenge (CEC 2019) numerical problems.

Problem		jDE100^71^	DE	PSO^72^	YDSE^73^	NMRA	PDO^40^	DHPN
*G*16	Mean	1.590E+05	5.864E+11	3.180E+05	1.836E+06	4.167E+05	**6.603E+04**	7.384E+04
	Std	1.597E+05	8.359E+11	4.470E+05	9.262E+05	2.857E+05	3.328E+04	**1.530E+04**
	p-rank	−	−	−	−	−	+	
	f-rank	3	7	4	6	5	1	2
*G*17	Mean	2.385E+06	6.844E+01	3.010E+02	**1.735E+01**	1.891E+01	1.794E+01	1.744E+01
	Std	2.719E+04	1.418E+02	9.820E+01	**4.300E-03**	5.381E-01	6.264E-01	1.024E-01
	p-rank	−	−	−	+	−	−	
	f-rank	7	5	6	1	4	3	2
*G*18	Mean	1.310E+06	**1.270E+01**	3.230E+00	**1.270E+01**	**1.270E+01**	**1.270E+01**	**1.270E+01**
	Std	8.519E+05	1.900E-03	1.860E+00	3.494E-05	4.552E-04	**9.596E-07**	3.102E-06
	p-rank	−	−	−	−	−	+	
	f-rank	7	5	6	3	4	1	2
*G*19	Mean	3.475E+05	1.539E+03	**3.160E+01**	5.389E+01	5.525E+02	9.631E+03	2.589E+03
	Std	1.149E+05	2.433E+03	1.280E+01	**1.037E+01**	5.488E+02	3.382E+03	1.679E+03
	p-rank	−	$+$	+	+	$+$	−	
	f-rank	7	4	1	2	3	6	5
*G*20	Mean	1.673E+05	2.116E+00	**1.810E+00**	2.170E+00	2.257E+00	3.737E+00	2.409E+00
	Std	8.426E+04	2.850E-01	**1.350E-01**	6.468E-01	5.225E-01	7.068E-01	4.828E-01
	p-rank	−	$+$	$+$	$+$	$+$	−	
	f-rank	7	2	1	3	4	6	5

*G*21	Mean	3.841E+04	9.901E+00	9.530E+00	1.007E+01	1.159E+01	8.777E+00	**8.496E+00**
	Std	2.063E+03	1.474E+00	1.180E+00	**6.818E-01**	7.990E-01	1.365E+00	7.371E-01
	p-rank	−	−	−	−	−	−	
	f-rank	7	4	3	5	6	2	1
*G*22	Mean	9.105E+06	1.282E+03	1.070E+03	2.658E+02	1.984E+02	6.800E+02	**3.003E+01**
	Std	4.38E+08	4.224E+02	3.210E+02	1.193E+02	1.612E+02	2.154E+02	**1.133E+02**
	p-rank	−	−	−	−	−	−	
	f-rank	7	6	5	3	2	4	1
*G*23	Mean	1.219E+09	6.982E+00	**4.290E+00**	6.007E+00	6.039E+00	6.094E+00	5.253E+00
	Std	4.388E+08	**2.255E-01**	3.010E-01	3.456E-01	4.872E-01	4.536E-01	4.528E-01
	p-rank	−	−	$+$	−	−	−	
	f-rank	7	6	1	3	4	5	2
*G*24	Mean	9.207E+08	2.788E+02	**3.210E+00**	6.172E+01	8.657E+00	1.239E+03	2.071E+02
	Std	1.131E+08	3.122E+02	4**.190E-01**	1.523E+02	2.002E+01	5.042E+02	1.865E+02
	p-rank	−	−	$+$	$+$	$+$	−	
	f-rank	7	5	1	3	2	6	4
*G*25	Mean	1.541E+06	2.042E+01	2.130E+01	2.039E+01	2.051E+01	2.032E+01	**2.007E+01**
	Std	7.460E+05	1.295E-01	1.040E-01	**1.034E-01**	1.367E-01	1.430E-01	5.646E-02
	p-rank	−	−	−	−	−	−	
	f-rank	7	4	6	3	5	2	1
w/l/t		0/10/0	2/8/0	4/6/0	4/6/0	3/7/0	2/8/0	NA
Average f-rank		6.60	4.80	3.40	3.20	3.90	3.60	2.50
Overall f-rank		7	6	3	2	5	4	1

### Convergence profiles

**Figure 2:**
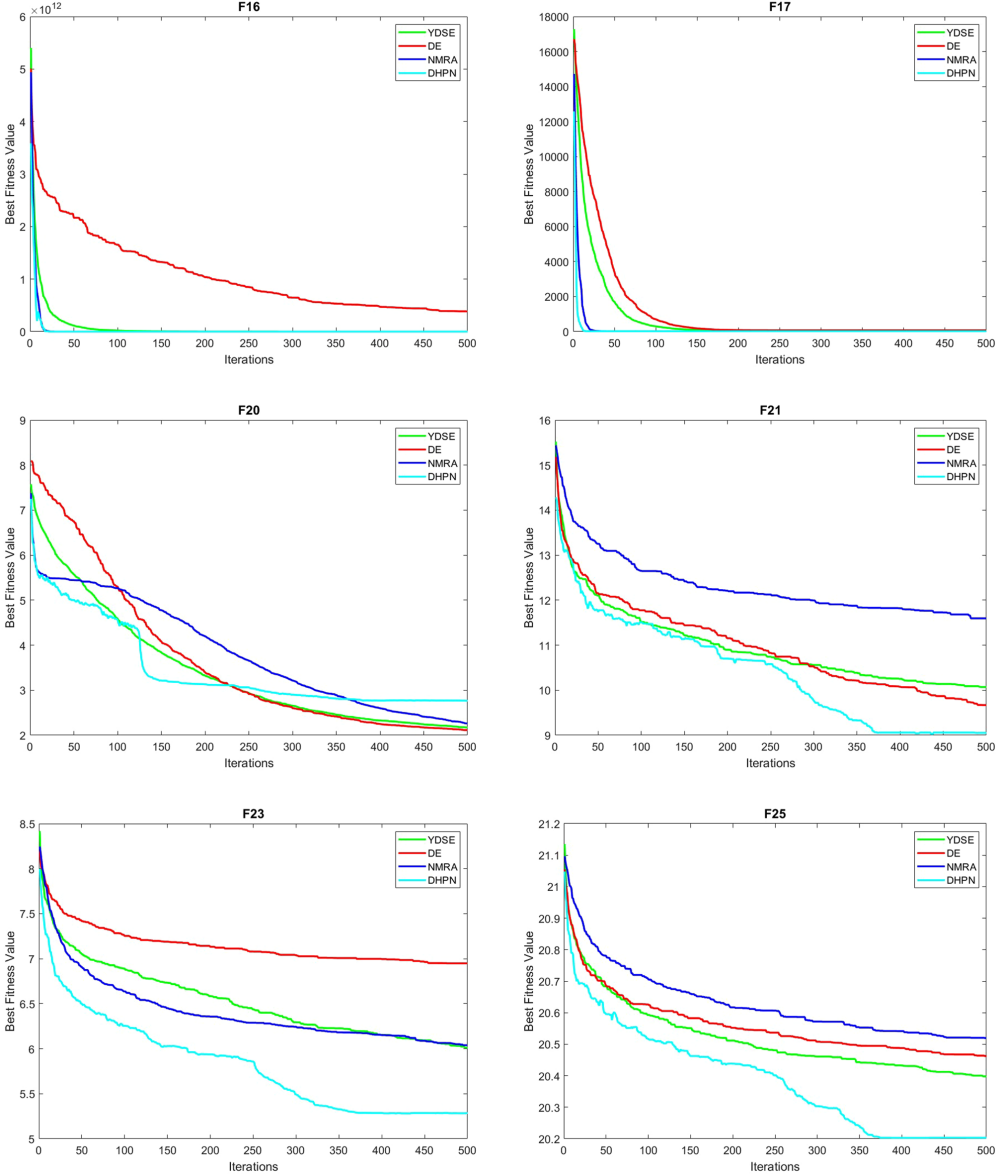
Convergence profiles of YDSE, DE, NMRA, and DHPN.

The convergence profiles of YDSE, DE, NMRA and the proposed DHPN algorithm are shown for functions *G*16, *G*17, *G*20, *G*21, *G*23 and *G*24 in Fig. 2. Here, convergence curves are drawn for maximum iterations, pop size and dimension as 500, 50 and 30, respectively. The convergence profiles for *G*16, *G*17, *G*21, *G*23 and *G*25 clearly confirm the fastest convergence of the DHPN algorithm among the other algorithms under comparison. For *G*20, the proposed DHPN algorithm begins to converge faster initially, but its rate of convergence decreases later. So, for *G*20, the convergence of DE is the fastest. Overall, the DHPN algorithm is superior to the existing algorithms in terms of convergence.

## Real world application: region-based image fusion using DHPN

**Figure 3:**
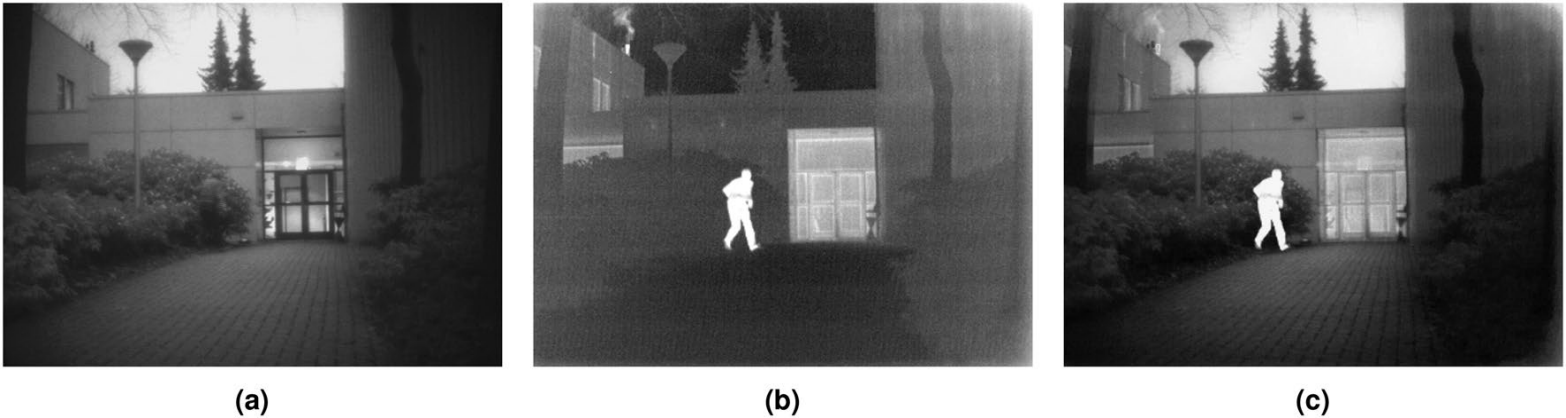
Example of an image pair obtained by different sensors and their fusion (**a**) VIS image (**b**) IR image and (**c**) Fused image.

The demand for image fusion in image processing applications has grown significantly with the tremendous increase in acquisition systems^45,46^. Image fusion is a process of integrating images acquired by various sensors to yield an informative image. An effective image fusion preserves vital information by extracting all essential properties and features from the images without resulting in any inconsistency in the fused image. This fused image is more apt for both human as well as machine perception as it gives information that cannot be obtained by examining several images individually. In certain applications, details from CCD and IR sensors are combined together with the objective of maintaining their distinct features^82^. The process of merging information obtained from several sensors is called MM image fusion^83,84^. An example of an image pair obtained from different sensors with their fused image is shown in Fig. 3. The final image clearly maintains the distinct features of both VIS and IR images. Human presence is depicted, and edges are preserved.

Over the last three decades, several image fusion techniques have been developed to improve image quality and make them appropriate for different applications such as remote sensing, target detection, surveillance, biological recognition, medical images, military field, computer vision, and robotics^85–87^.

### Related work

#### Image fusion

The image fusion techniques can be categorized into pixel level, decision level and feature level. Pixel-level image fusion techniques integrate the meaningful data from source images at the lowest physical level directly^88^. Feature-level image fusion techniques involve the extraction of relevant features like textures, edges or pixel intensities, which are then combined to form merged images^53,89^. Decision-level image fusion is the most complex and highest-level fusion. In this, decisions made from initial object detection and classification tasks are merged^90^. As compared to feature level and decision level techniques, pixel-level image fusion provides the maximum amount of details and is more suitable for applications that are dependent on different sensors^91^.

The pixel-level techniques can be classified as substitution techniques and transform domain techniques. Substitution methods include Independent Component Analysis (ICA)^92^, Principal Component Analysis (PCA)^93^, whereas transform domain techniques include Pyramid Transform (PT)^94^, Discrete Wavelet Transform (DWT)^95^, shift-variant shearlet transform^96^, curvelet^54^, and contourlet transform^97^. Although the aforementioned methods have less computation cost, however, they possess noise and are unable to preserve adequate details of source images^98^.

#### Visible and infrared image fusion

IR images are more robust to varying light levels and environmental deterioration. On the contrary, VIS images have finer visual quality with precise background information like texture and higher pixel pitch. The objective of fusing VIS and IR images is to derive significant details from both images and incorporate them in the compound image^47^. Hence, over the past two decades, VIS and IR image fusion has become an intensive area of research in multi-band nighttime monitoring and navigation imagery.

The most widely used methods for fusing VIS and IR images are region-based techniques^99,100^. Region-based image fusion techniques include the segmentation of source images into a number of segments or clusters on the basis of region-based algorithms and merging them region by region^92,99,101^. The most significant stage of such techniques is the extraction of salient details from IR images. The present work employs a novel optimization strategy for region-based image fusion of VIS and IR images.

In the proposed region-based technique, DHPN is used to segment the VIS image to derive its salient features. The extracted features are used to calculate the weight map function of both VIS and IR images. These weight map functions are further used to form the final fused image.

#### Multi-level thresholding for image segmentation

Image segmentation is a pivotal pre-processing task in image processing. An effective, simple way to achieve image segmentation is through image thresholding. It is capable of classifying pixels into different groups through a set of threshold levels^55,102^. The aim of thresholding is to compute the value of optimal threshold levels for differentiating the target from its background^103^. If a method chooses only two optimum threshold levels, then it is said to be a bi-level or two-level thresholding method. On the other hand, in a multi-level thresholding, an image is segmented on the basis of multiple optimum threshold levels.

The widely used thresholding techniques are mainly categorized as parametric and non-parametric techniques. Parametric techniques are computationally expensive in terms of both cost and time, as these methods require the estimation of Probability Density Functions (PDFs). On the other hand, non-parametric techniques yield results with better accuracy and less computational cost^104^. Such techniques calculate threshold values by measurement of entropy^105^, error rate, between-class variance^106^, local maxima, and so on. The most preferred method for thresholding-based image segmentation is the OTSU method^106^.

### Image segmentation using OTSU method

Also known as the Between-class variance method, the OTSU method is an adaptive binarization thresholding algorithm that was proposed by Japanese scholar OTSU in 1979. This non-parametric, unsupervised method utilizes between-class variance for separating the segmented classes. It obtains a threshold value that maximizes inter-class variance, resulting in minimum intra-class variance between pixel intensities of each class. Thus, this method divides a given image into background and foreground depending on its gray-scale characteristics, such that the difference between them is the largest with the best threshold.

The fundamental principle of image segmentation using OTSU method is described below.

Consider the gray scale range of an image is $k=0,1,2,...,K-1$. If $m_i$ signifies the amount of pixels with *i*th gray level, then the total pixels *M* in the image are

59 $$\begin{aligned} M=\sum _{i=0}^{K-1}m_i=m_0+m_1+m_2+...+m_{K-1}. \end{aligned}$$

The likelihood of occurrence of *i*th gray level is given by

60 $$\begin{aligned} p_i=\frac{m_i}{M}=\frac{m_i}{\sum _{i=0}^{K-1}m_i}. \end{aligned}$$

Assuming *T* is the gray level threshold which divides the image into two classes: $Y_0=\{0,1,...,T\}$ and $Y_1=\{T+1,T+2,...,K-1\}$, the probability of both $Y_0$ and $Y_1$ are

61 $$\begin{aligned} \Omega _0=\sum _{i=0}^{T}p_i \end{aligned}$$

and

62 $$\begin{aligned} \Omega _1=\sum _{i=T+1}^{K-1}p_i, \end{aligned}$$

The means and variances of both $Y_0$ and $Y_1$ are

63 $$\begin{aligned}{} & {} \mu _0=\sum _{i=0}^{T}\frac{ip_i}{\Omega _0}, \end{aligned}$$

64 $$\begin{aligned}{} & {} \mu _1=\sum _{i=T+1}^{K-1}\frac{ip_i}{\Omega _1}, \end{aligned}$$

65 $$\begin{aligned}{} & {} \sigma _0^2=\sum _{i=0}^{T}(i-\mu _0)^2p_i/\Omega _0 \end{aligned}$$

and

66 $$\begin{aligned} \sigma _1^2=\sum _{i=T+1}^{K-1}(i-\mu _1)^2p_i/\Omega _1, \end{aligned}$$

If $\mu _T$ denotes mean intensity of the image, then

67 $$\begin{aligned} \Omega _0 \mu _0+\Omega _1 \mu _1=\mu _T \end{aligned}$$

and

68 $$\begin{aligned} \Omega _0+\Omega _1=1. \end{aligned}$$

The between-class variance and inter-class variance are defined as

69 $$\begin{aligned} \sigma _B^2=\Omega _0(\mu _0-\mu _T)^2+\Omega _1(\mu _1-\mu _T)^2. \end{aligned}$$

and

70 $$\begin{aligned} \sigma _I^2=\Omega _0\sigma _0^2+\Omega _1\sigma _1^2, \end{aligned}$$

Hence, the total variance is

71 $$\begin{aligned} \sigma _T^2=\sigma _I^2+\sigma _B^2 \end{aligned}$$

To perform thresholding, the OTSU method maximizes the between-class variance, which minimizes the inter-class variance as the total variance is constant. This method can be extended to multi-level thresholding by assuming $N-1$ thresholds $\{T_1,T_2,...,T_{N-1}\}$ that segment the total pixels into *N* different classes $\{Y_0,Y_1,...,Y_{N-1}\}$. The thresholds can be calculated by

72 $$\begin{aligned} \begin{aligned} \{T_1,T_2,...,T_{N-1}\}&=\arg \{\max _{0\le T\le K-1}\{ \sigma _B^2 (T_1,T_2,...,T_{N-1})\}\}\\&=\arg \{\min _{0\le T\le K-1}\{ \sigma _I^2 (T_1,T_2,...,T_{N-1})\}\} \end{aligned} \end{aligned}$$

where

73 $$\begin{aligned}{} & {} \Omega _j=\sum _{i=T_{j-1}+1}^{T_j}p_i, \end{aligned}$$

74 $$\begin{aligned}{} & {} \mu _j=\sum _{i=T_{j-1}+1}^{T_j}ip_i/\Omega _j, \end{aligned}$$

75 $$\begin{aligned}{} & {} \sigma _j^2=\sum _{i=T_{j-1}+1}^{T_j}(i-\mu _j)^2p_i/\Omega _j, \end{aligned}$$

76 $$\begin{aligned}{} & {} \sigma _B^2=\sum _{j=1}^N\Omega _j(\mu _j-\mu _T)^2 \end{aligned}$$

and

77 $$\begin{aligned} \sigma _I^2=\sum _{j=1}^N\Omega _j\sigma _j^2. \end{aligned}$$

### Weight map computation and fusion strategy

The proposed DHPN algorithm calculates the threshold values of an input image to obtain the segmented image along with its weight map. Assuming the input image to be an IR image, then the corresponding segmented image and its weight map are given by,

78 $$\begin{aligned} I_{SI}(a,b)=\text {DHPN}(I_{IR}(a,b)) \end{aligned}$$

and

79 $$\begin{aligned} W_{IR}^1(a,b)=I_{SI}(a,b), \end{aligned}$$

The weight map of VIS image corresponding to the input IR image is

80 $$\begin{aligned} W_{VIS}^1(a,b)=\max ( W_{IR}^1(a,b))- W_{IR}^1(a,b). \end{aligned}$$

The weight maps $W_{IR}^1$ and $W_{VIS}^1$ are hard and noisy. Thus, they are unsuitable for the fusion of input images. In particular, IR images include coarse-scale structural data. While VIS images depict fine-scale structures. Fusing them directly may result in more insignificant details from IR images and fine-scale details from VIS images. To remove these artefacts in the final fused image, the WLS^107^ optimization scheme is used to refine both weight maps.

WLS preserves edges by striking a balance between sharpening and blurring. Hence, it is an edge-preserving and smoothing filter that progressively sharpens an image whilst preserving its spacial information^107^. For weight maps $W_{IR}^1$ and $W_{VIS}^1$, the corresponding refined weights $W_{IR}^2$ and $W_{VIS}^2$ can be found by minimizing functions $F_{IR}$ and $F_{VIS}$, respectively.

81 $$\begin{aligned}{} & {} F_{IR}=\sum _p \left( \left( W_{(IR)p}^2- W_{(IR)p}^1\right) ^2+\lambda \left( W_{a,p}W_{IR}^1\left( \frac{\partial W_{IR}^2}{\partial a}\right) _p^2+W_{b,p}W_{IR}^1\left( \frac{\partial W_{IR}^2}{\partial a}\right) _p^2\right) \right) \end{aligned}$$

82 $$\begin{aligned}{} & {} F_{VIS}=\sum _p \left( \left( W_{(VIS)p}^2- W_{(VIS)p}^1\right) ^2+\lambda \left( W_{a,p}W_{VIS}^1\left( \frac{\partial W_{VIS}^2}{\partial a}\right) _p^2+W_{b,p}W_{VIS}^1\left( \frac{\partial W_{VIS}^2}{\partial a}\right) _p^2\right) \right) \end{aligned}$$

After obtaining refined weight maps $W_{IR}^2$ and $W_{VIS}^2$, the pixel-wise single-scale weighted average composition is carried out to get the fused image, $I_{FI}$ as

83 $$\begin{aligned} I_{FI}(a,b)=W_{IR}^2(a,b)\times I_{IR}(a,b)+W_{VIS}^2(a,b)\times I_V(a,b) \end{aligned}$$

### Experimental results

This section demonstrates the effectiveness of the DHPN algorithm in the context of pixel-based image fusion. The DHPN algorithm is analysed against the existing image fusion algorithms based on discrete cosine harmonic wavelet (DCH)^108^, cross bilateral filter (CBF)^109^, gradient transfer method (GTF)^110^. Saliency detection in sparse domain (JSR)^111^, convolutional sparse representation (CSR)^112^ and deep convolutional neural network (CNN)^113^. For simulations, twelve pairs of visible and infrared images are chosen from TNO data set^114^ which is available online at https://doi.org/10.6084/m9.figshare.1008029.v1. Further, comparative analysis has been done on the basis of four fusion metrics, namely edge-based similarity index ($Q^{AB/F}$), the sum of correlation difference (SCD), structural similarity index measure (SSIM) and artefact measure ($N^{AB/F}$), which are discussed in the subsequent subsections. Also, their MATLAB codes are available in the public domain.

#### Edge based similarity index ($Q^{AB/F}$)

This index represents the edge preservation detail in the fused images with a range between (0, 1). It can be calculated as^115,116^

84 $$\begin{aligned}{} & {} Q^{AB/F}=\frac{\sum _{u=1}^U\sum _{v=1}^V Q^{A/F}(u,v)q_A(u,v)+Q^{B/F}(u,v)q_B(u,v)}{\sum _{u=1}^U\sum _{v=1}^V (u,v)q_A(u,v)+(u,v)q_B(u,v)}, \end{aligned}$$

85 $$\begin{aligned}{} & {} Q^{A/F}(u,v)=Q_q^{A/F}(u,v) Q_o^{A/F}(u,v), \end{aligned}$$

86 $$\begin{aligned}{} & {} Q^{B/F}(u,v)=Q_q^{B/F}(u,v) Q_o^{B/F}(u,v), \end{aligned}$$

where *F* represents the fused image of source images *A* and *B*; $q_A(u,v)$ and $q_B(u,v)$ denote weights for images *A* and *B* at pixel position (*u*, *v*), respectively. The parameters $Q_o^{A/F}$ and $Q_o^{B/F}$ represent preservation of orientation values, whereas the parameters $Q_q^{A/F}$ and $Q_q^{B/F}$ denote strength of edge in images *A* and *B* at pixel location (*u*, *v*), respectively.

The $Q^{AB/F}$ values of different algorithms are given in Table 10. Ideally, the value of $Q^{AB/F}$ should be one, indicating that all the edge details are preserved. It is clearly evident that the $Q^{AB/F}$ value obtained in the case of the proposed DHPN algorithm is 0.765508, which is higher than that of the other algorithms for all twelve pairs. This signifies that the proposed algorithm preserves edge details in the fused images quite significantly with respect to the existing algorithms.

**Table 10:** Comparison of various algorithms on the basis of $Q^{AB/F}$.

Image set	Algorithm						
	DCH	CBF	GTF	JSR	CNN	CSR	DHPN
Camp	0.39101	0.392	0.40514	0.30318	0.38339	0.47638	**0.7809**
Traffic	0.47003	0.45063	0.37349	0.5899	0.33248	0.5422	**0.8041**
Building	0.60936	0.54724	0.5382	0.3571	0.42222	0.64338	**0.8122**
Home post	0.48	0.47643	0.38912	0.28404	0.27618	0.53147	**0.7912**
Bench	0.63058	0.63068	0.57943	0.38013	0.42865	0.66955	**0.8924**
Bunker	0.57579	0.59259	0.48723	0.30221	0.24415	0.56803	**0.726**
Heather	0.42805	0.40948	0.46613	0.19807	0.18651	0.47147	**0.4924**
Light hut	0.43398	0.43149	0.39907	0.38772	0.3491	0.52489	**0.7879**
Lake	0.53952	0.53923	0.48131	0.2751	0.21755	0.54689	**0.7703**
Doorway	0.50494	0.50734	0.47549	0.29007	0.32607	0.5424	**0.7445**
Soldier	0.45498	0.40809	0.41948	0.26842	0.15541	0.56881	**0.7386**
Trench	0.60639	0.59442	0.55742	0.28463	0.23039	0.61256	**0.8456**
Average	0.510386	0.498302	0.464293	0.326714	0.296008	0.558169	**0.765508**

Significant values are in bold.

#### Sum of correlation difference (SCD)

SCD denotes the extent of useful information that is transmitted to the fused images from its corresponding source images^99,117^. For good fusion results, a higher value of SCD is desirable. It is computed as

87 $$\begin{aligned} SCD=corr(diff_1,A)+corr(diff_2,B), \end{aligned}$$

where $diff_1=F-B$, $diff_2=F-A$ and *corr*(.) represents correlation function.

The SCD values for various algorithms are given in Table 11. The results show the superiority of the DHPN algorithm over other algorithms for five image sets in terms of SCD values. On the contrary, CSR is best for four image sets, JSR is best for two image sets, and DCH yields a higher value of SCD for one image set only. Overall, the proposed DHPN algorithm gives a higher average value of SCD, which is desirable. This shows that with DHPN, more meaningful data is transmitted into the final fused image than with the existing algorithms.

**Table 11:** Comparison of various algorithms on the basis of SCD.

Image set	Algorithm						
	DCH	CBF	GTF	JSR	CNN	CSR	DHPN
Camp	1.44541	1.30442	0.96895	1.4111	1.33851	1.51433	**1.605**
Traffic	1.43676	1.27191	1.10568	1.70293	1.31964	1.54966	**1.8013**
Building	1.60591	1.4797	0.97451	1.58464	0.91136	1.71489	**1.7441**
Home post	1.71128	1.39012	1.05256	1.77756	1.54743	**1.77825**	1.7672
Bench	1.81492	1.62827	1.08473	1.79545	1.71422	1.68696	**1.8669**
Bunker	1.56726	1.36803	1.15649	**1.62841**	1.51574	1.50995	1.5467
Heather	1.6286	1.39251	1.19997	1.46388	0.96053	**1.64955**	1.2101
Light hut	**1.65352**	1.45965	0.92405	1.54558	1.52093	1.62265	1.6283
Lake	1.60896	1.36132	1.17275	**1.70845**	1.66239	1.67671	1.7048
Doorway	1.68888	1.45324	1.11126	1.61929	1.44563	**1.71431**	1.5208
Soldier	0.94824	0.53538	0.73046	1.34397	1.43624	**1.47326**	1.4505
Trench	1.60723	1.20809	0.97403	1.73614	1.46113	1.6444	**1.7365**
Average	1.559748	1.321053	1.037953	1.609783	1.402813	1.62791	**1.63185**

Significant values are in bold.

#### Structural similarity index measure (SSIM)

As the name suggests, SSIM represents the extent of similarity between two images with a range between (0, 1). It is preferable when the ground truth is available. However, in the case of image fusion, SSIM is calculated as

88 $$\begin{aligned} SSIM(A,F)=\frac{(2\alpha _A\alpha _F+C_1)(2\sigma _{A/F}+C_2)}{(\alpha _A^2+\alpha _F^2+C_1)(\sigma _A^2+\sigma _F^2+C_2)}, \end{aligned}$$

89 $$\begin{aligned} SSIM(B,F)=\frac{(2\alpha _B\alpha _F+C_1)(2\sigma _{B/F}+C_2)}{(\alpha _B^2+\alpha _F^2+C_1)(\sigma _B^2+\sigma _F^2+C_2)} \end{aligned}$$

and

90 $$\begin{aligned} SSIM= \frac{SSIM(A,F)+SSIM(B,F)}{2}. \end{aligned}$$

Here, $\alpha _A$, $\alpha _B$ and $\alpha _F$ denote mean intensities, whereas $\sigma _A^2$, $\sigma _B^2$ and $\sigma _F^2$ represent variance of images *A*, *B*, and *F*, respectively. The parameters $\sigma _{A/F}$ and $\sigma _{B/F}$ signify covariance of source images *A* and *B*, and fused image *F*, respectively. $C_1$ and $C_2$ denote constants.

Table 12 gives SSIM values for twelve pairs of images using various algorithms. Higher the value of SSIM, higher is the similarity of constituent images with the fused image. It is quite evident from the results that out of twelve images, CSR performs best for three image sets, whereas DHPN outperforms other existing image fusion algorithms for nine image sets. Overall, DHPN gives the highest value 0.726317 of SSIM among all the existing. Hence, DHPN performs superiorly in terms of average SSIM value.

**Table 12:** Comparison of various algorithms on the basis of SSIM.

Image set	Algorithm						
	DCH	CBF	GTF	JSR	CNN	CSR	DHPN
Camp	0.74834	0.62376	0.69181	0.52715	0.70536	**0.74954**	0.7103
Traffic	0.64468	0.49861	0.61109	0.62353	0.64394	**0.67474**	0.6632
Building	0.77224	0.58315	0.76132	0.59028	0.764	**0.81199**	0.8
Home post	0.73449	0.61724	0.73213	0.50549	0.71112	0.76172	**0.7725**
Bench	0.57614	0.5236	0.50273	0.45458	0.55761	0.56129	**0.5923**
Bunker	0.65663	0.61793	0.62984	0.4725	0.62314	0.66693	**0.6936**
Heather	0.70979	0.63041	0.67283	0.44712	0.6714	0.72858	**0.7508**
Light hut	0.70607	0.53699	0.65676	0.56569	0.66818	0.71927	**0.7206**
Lake	0.75918	0.69888	0.74249	0.52336	0.7315	0.78059	**0.7913**
Doorway	0.6883	0.59021	0.67176	0.48033	0.66418	0.71294	**0.7308**
Soldier	0.62304	0.53005	0.62966	0.57412	0.65703	**0.70304**	0.6996
Trench	0.74211	0.68207	0.73201	0.52062	0.7277	0.77933	**0.7908**
Average	0.696751	0.594408	0.669536	0.523731	0.677097	0.72083	**0.726317**

Significant values are in bold.

#### Artefact measure ($N^{AB/F}$)

The process of image fusion may add some meaningless visual details to the final fused image. These artefacts are undesirable and could lead to misinformation which can severely affect fusion applications. The measure $N^{AB/F}$^118,119^ gives the extent of such distortion or noise introduced in the final merged image, and hence its lower value is preferable.

The values of $N^{AB/F}$ for different image fusion methods are given in Table 13. It is evident that the value of $N^{AB/F}$ is minimum for eleven image sets in the case of the proposed DHPN algorithm, whereas the CSR algorithm yields the lowest value for only one image set, ”Camp”. Overall, DHPN gives a minimum average $N^{AB/F}$ value, i.e. 0.006617, as compared to the existing algorithms. This signifies that DHPN introduces the least amount of artefacts in the output image of all the other image fusion algorithms.

**Table 13:** Comparison of various algorithms on the basis of $N^{AB/F}$.

Image set	Algorithm						
	DCH	CBF	GTF	JSR	CNN	CSR	DHPN
Camp	0.05118	0.23167	0.07027	0.34153	0.02976	**0.01494**	0.0457
Traffic	0.2184	0.487	0.11237	0.19889	0.05862	0.02199	**0.002**
Building	0.19093	0.45288	0.143	0.42353	0.03609	0.02378	**0.0081**
Home post	0.10268	0.23932	0.06399	0.36509	0.02096	0.02296	**0.0022**
Bench	0.05781	0.15233	0.12329	0.21536	0.03424	0.03404	**0.0142**
Bunker	0.05342	0.11741	0.09009	0.30761	0.0077	0.02371	**0.000309**
Heather	0.09272	0.2009	0.10404	0.34271	0.02796	0.02013	**0.000821**
Light hut	0.09951	0.36066	0.06577	0.2822	0.05044	0.01888	**0.000974**
Lake	0.08579	0.18971	0.09124	0.40261	0.01751	0.02037	**0.0011**
Doorway	0.09529	0.21509	0.0752	0.35013	0.02381	0.02207	**0.0012**
Soldier	0.24507	0.25892	0.09293	0.16541	0.02271	0.02574	**0.000703**
Trench	0.13342	0.18091	0.12682	0.38546	0.01798	0.02746	**0.0021**
Average	0.118852	0.257233	0.096584	0.315044	0.028982	0.023006	**0.006617**

Significant values are in bold.

**Figure 4:**
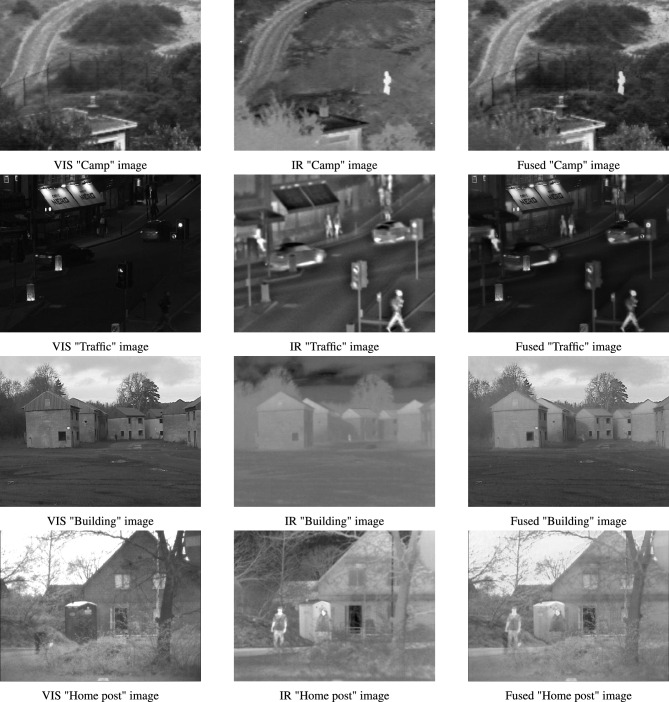

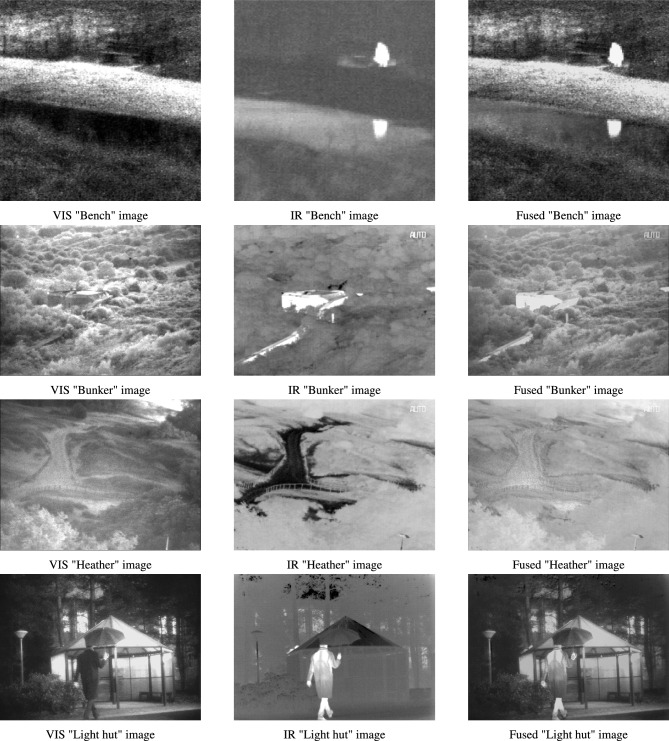

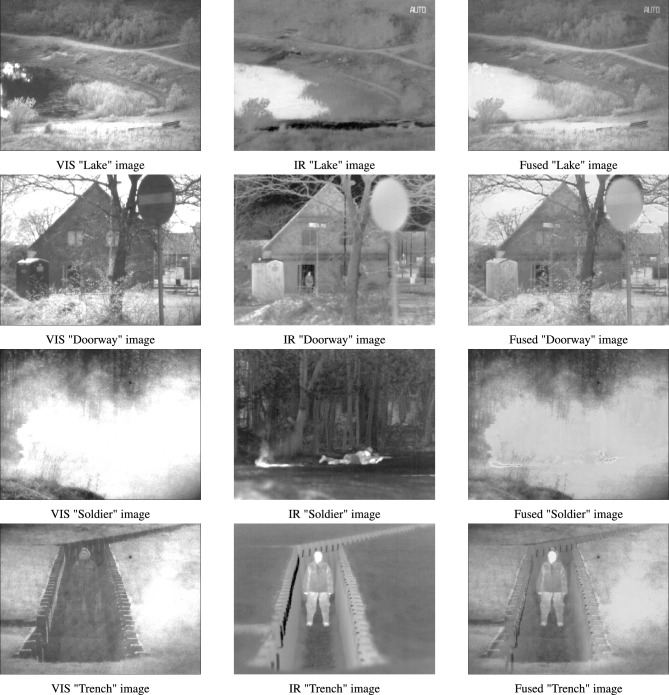
Image fusion results using proposed DHPN algorithm.

Furthermore, fused images of various image sets using the DHPN algorithm are given in Fig. 4. In image sets ”Camp”, ”Traffic”, ”Building”, ”Home post”, ”Bench”, ”Doorway”, ”Soldier” and ”Trench”, a human figure is visible only in the corresponding IR images, whereas background details like buildings, foliage, trees can be seen in the corresponding VIS images. DHPN combines both of these features in the corresponding fused images, thus, increasing the information content. For image sets ”Bunker”, ”Heather”, ”Light hut” and ”Lake”, the objective is to insert spectral details present in the IR images into the corresponding VIS images for night-vision context enhancement. It can be seen that the corresponding fused images show better context enhancement and have details from both the VIS and IR images. In a nutshell, DHPN preserves background details from VIS images and object details from IR images in the corresponding final fused images. Thus, DHPN is capable of transferring necessary details from VIS as well as IR images into the fused images. This is also confirmed by the comparative analysis based on different fusion quality metrics.

## Discussion

This section has three subsections, namely summary, which highlights the major details of the proposed algorithm, its application to benchmark and image fusion. The second subsection provides the drawbacks of the proposed approach, and in the final subsection, we provide some insightful implications.

### Summary of results

- The proposed DHPN consists of a balanced *expl* phase by incorporating DMO, HBA, and PDO into NMRA. A stagnation phase is incorporated for local optima avoidance, and finally, six new *iws* (simulated annealing, exponential, linear, chaotic, oscillating, and random) are added to make it self-adaptive.
- In order to evaluate the performance, a pop size and dimension size comparison is done. To check for the best set of parameters, an analysis of parameters is also presented. It has been found that if the pop size is reduced, the algorithm diversity reduces, and hence, the results are not that significant. However, when we increase the pop size, the variation in results is very limited. So it can be said that a pop size of 50 can be considered the best. The same is true for dimension size, where the results do degrade over higher dimensions, but there are no significant changes in results. Hence, making the algorithm a better fit for higher dimensions as well.
- These operators have been applied to all the parameters of DHPN to make it adaptive in nature. These parameter adaptations help the algorithm in self-tuning, with no requirement for adaptation from the user perspective.
- All of these added advantages are tested over CEC 2005 as well as CEC 2019 benchmarks. It has been found that DHPN provides good optimal results in comparison to other algorithms in the literature.
- For CEC 2005, the proposed algorithm scores the first rank, HBA has the second rank, and JADE ranks third. For CEC 2019, DHPN was also ranked first, and YDSE was the second-best algorithm.
- Further, DHPN is applied to the real-world problem of region-based image fusion of VIS and IR images. The average $Q^{AB/F} = 0.765508$, $SCD = 1.63185$, $SSIM = 0.726317$, and $N^{AB/F} = 0.006617$ shows the best combination of results obtained by DHPN with respect to other algorithms such as DCH, CBF, GTF, JSR and others. Experimental tests showed that the proposed algorithm provides reliable results.

### Drawbacks

- A critical issue with the algorithm is the use of a highly intensive *expl* combined with *expt* operation in the worker phase. Thus making the algorithm more of an exploitative one, and hence increasing the chances of poor *expl*.
- It’s true that the stochastic nature of algorithms can lead to getting stuck in local optima, and there’s no guarantee of reaching a global solution. This limitation applies to many stochastic optimization algorithms, including the proposed DHPN algorithm. While DHPN may perform well for certain types of problems, it may not be suitable for others, where finding the global optimum is crucial. It’s important to carefully consider the characteristics of the problem at hand and assess whether the stochastic nature of DHPN aligns with the problem’s requirements. Additionally, employing strategies such as hybridization or incorporating problem-specific knowledge may help mitigate this limitation and improve the algorithm’s performance across a wider range of problems.
- A balance between *expl* versus *expt* is also not guaranteed in the proposed algorithm. This is because of the presence of a more enhanced *expl* operation rather than *expt* operation. Thus, an extensive experimental study is required on how we can achieve this effectively.

### Insightful implications

- The proposed DHPN is easier to implement and can be applied to hybrid and expert intelligent systems. We can further enhance its *expl* and *expt* operations and can make it the best fit for real-world problems.
- The algorithm can be changed to a binary version for use in medical imaging, wireless sensor networks, electroencephalogram, clustering and other binary domain problems.
- DHPN can also be extended to multiple engineering design problems, including multi-objective optimization problems for solving problems with two to many objectives.
- Apart from these, the algorithm can be explored more to make all its parameters auto-tunable, whereby auto-tunable means that with the progression of the iterations, the algorithm will automatically tune its parameters to fit the problem under consideration.

## Conclusion and future work

This paper presents an extensive study of a new multiple hybrid algorithm formulated using four algorithms, including DMOA, HBO, PDO, and NMRA. The algorithm has a new stagnation phase and self-adaptive mutation operators for local optima avoidance and parametric enhancements. The DHPN algorithm is checked for CEC 2005 and CEC 2019 problems for performance evaluation. A deeper analysis of the population and dimension size shows that the proposed algorithm fares significantly better for medium pop sizes and can provide good results for higher dimensional problems. A comparative study with JADE, OEWOA, SaDE, FA-FPO, GWO-E, SHADE, SCCSA, CMA-ES, LSHADE-SPACMA, and jDE100, among others, shows that the proposed DHPN provides highly reliable results. Even from the statistical results of Wilcoxon and Friedman tests, DHPN has been found to provide better results. Furthermore, DHPN is used as an optimization strategy in region-based image fusion. The efficacy and efficiency of DHPN in image fusion are proven with respect to the four fusion quality metrics.

However, the proposed algorithm has the extensive *expl* operation, but suffers from the poor *expt* operation. A balance between both operations is desirable. For future results, a balance between the *expl* and *expt* operation must be performed. For parametric adaptations, we can explore various other mutation/inertia wight operations, including trigonometric, Gaussian, Cauchy and others. New mathematical equations can be added to further improve the *expl* and *expt* operations. A detailed theoretical and numerical study can be added to see how the algorithm behaves theoretically. Further, as an extension, DHPN can be applied to gene expression modelling, cancer classification, feature selection, and clustering problems, among others.

Some other future works include the exploration of hybrid approaches, particularly integrating DHPN with other optimization techniques and domain-specific algorithms. Advanced versions of DHPN can be designed for handling multi-objective and many-objective optimization problems, adapting to dynamic environments, and scaling for large-scale and parallel optimization. Additionally, the intersection of DHPN with quantum computing is also expected to lead to breakthroughs in evolutionary quantum algorithms, revolutionizing optimization capabilities. Overall, the future of DHPN is characterized by continued innovation, driving its broader adoption and impact across diverse domains.

## Data Availability

The datasets used and/or analysed during the current study are available from the second corresponding author (Amanjot Kaur Lamba) on reasonable request.
